# Nanodrugs Targeting Key Factors of Ferroptosis Regulation for Enhanced Treatment of Osteoarthritis

**DOI:** 10.1002/advs.202412817

**Published:** 2025-01-22

**Authors:** Dong Wang, Yanli Pan, Wenzhe Chen, Du He, Weihui Qi, Jiali Chen, Wenhua Yuan, Yimin Yang, Di Chen, Pinger Wang, Hongting Jin

**Affiliations:** ^1^ Institute of Orthopaedics and Traumatology The First Affiliated Hospital of Zhejiang Chinese Medical University (Zhejiang Provincial Hospital of Chinese Medicine) Department of Orthopedic Surgery Hangzhou Hospital of Traditional Chinese Medicine Zhejiang Chinese Medical University Hangzhou 310000 China; ^2^ Faculty of Pharmaceutical Sciences Shenzhen University of Advanced Technology Shenzhen 518107 China

**Keywords:** cartilage, ferroptosis, multitarget regulation, nanoparticles, osteoarthritis

## Abstract

Osteoarthritis (OA) is a globally prevalent degenerative joint disease. Recent studies highlight the role of ferroptosis in OA progression. Targeting ferroptosis regulation presents a promising therapeutic strategy for OA; however, current research primarily focuses on single targets associated with ferroptosis. In this study, a reactive oxygen species (ROS)‐responsive nanoparticle is developed by linking deferasirox (DEF) and pterostilbene (PTE) with thioketal and incorporating cerium ions (Ce), creating Ce@D&P nanoparticles (NPs), which offer multitarget regulation of ferroptosis. The characteristics of Ce@D&P NPs are evaluated and their therapeutic effects on IL‐1β‐stimulated chondrocytes are verified. Results show that Ce@D&P NPs reduce ROS levels, mitigate inflammation, chelate iron to inhibit ferroptosis, and balance extracellular matrix (ECM) metabolism in chondrocytes. Mechanistically, transcriptomics and metabolomics analyses suggest that Ce@D&P NPs exerted their effects by regulating oxidative stress and lipid metabolism in chondrocytes. To better treat destabilization of the medial meniscus (DMM)‐induced OA in mice, Ce@D&P NPs via intra‐articular injection are delivered. The results show that Ce@D&P NPs alleviate cartilage matrix damage and slow OA progression. Overall, the findings indicate that Ce@D&P NPs represent a promising multitarget drug delivery system, and Ce@D&P NPs may be an effective strategy for OA treatment.

## Graphical abstract

Osteoarthritis is a common joint disease globally, bringing heavy social and economic burdens. Ferroptosis in OA, due to inflammation or iron overload, is a key factor. In this study, Ce@D&P nanoparticles (NPs) are developed to perform multitarget regulation of ferroptosis. Ce@D&P NPs can reduce ROS, relieve inflammation, inhibit ferroptosis, and balance extracellular matrix metabolism in chondrocytes and in destabilization of the medial meniscus‐induced OA mice, which may provide a new and effective treatment option for OA.

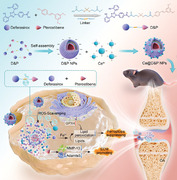

## 1 Introduction

Osteoarthritis (OA) is a prevalent degenerative joint disease, affecting ≈240 million people globally.^[^ ^1^ ^]^ The primary pathological features of OA include the loss of joint cartilage extracellular matrix (ECM), synovial inflammation, and abnormal subchondral bone remodeling.^[^ ^2^ ^]^ Despite previous studies indicating that the loss of cartilage ECM and chondrocyte death are critical mechanisms in OA pathogenesis, effective treatment strategies to slow cartilage degeneration have not been developed.^[^ ^3^, ^4^, ^5^ ^]^ Thus, exploring new therapeutic strategies is essential.

Recent research has revealed a close relationship between ferroptosis and OA progression, suggesting that chondrocyte ferroptosis is a significant factor in OA‐related cartilage degeneration.^[^ ^3^, ^6^ ^]^ Ferroptosis is a novel form of programmed cell death distinct from necrosis, apoptosis, and autophagy.^[^ ^7^ ^]^ The primary mechanism of ferroptosis involves intracellular iron overload, which, in combination with reactive oxygen species (ROS), triggers the Fenton reaction, catalyzing lipid peroxidation of the cell membrane and ultimately leading to cell death.^[^ ^8^ ^]^ OA exhibits characteristics similar to ferroptosis, such as abnormal intra‐articular iron metabolism,^[^ ^9^ ^]^ elevated ROS expression, enhanced inflammatory responses,^[^ ^10^ ^]^ and the involvement of lipid peroxidation derivatives in catabolic and proinflammatory processes within OA.^[^ ^11^ ^]^ Previous studies have shown that chondrocytes undergo ferroptosis under conditions of inflammation and iron overload.^[^ ^6^ ^]^ Chondrocyte ferroptosis exacerbates metabolic imbalances in joint cartilage, leading to increased expression of matrix metalloproteinase 13 (MMP13), degradation of ECM components like aggrecan (ACAN) and collagen‐II (Col‐2), accelerating OA progression.^[^ ^12^ ^]^ Recent studies indicate that inhibiting intracellular iron concentration and lipid ROS in chondrocytes can mitigate OA progression.^[^ ^13^, ^14^ ^]^ Therefore, regulating iron homeostasis and ROS levels to inhibit chondrocyte ferroptosis may be an effective strategy for slowing OA. However, current ferroptosis inhibitors mainly target single molecules associated with ferroptosis, such as ferrostatin 1 (Fer‐1), which inhibits lipid peroxidation of cell membranes. Fer‐1′s low biological stability limits its clinical application.^[^ ^15^ ^]^ Thus, developing clinically applicable multitarget strategies to inhibit ferroptosis is necessary.

Deferasirox (DEF), an iron chelator that binds trivalent iron ions, is a well‐known ferroptosis inhibitor.^[^ ^16^, ^17^ ^]^ Previous studies have reported DEF's therapeutic effects in diseases like ulcerative colitis and ischemia/reperfusion kidney injury through ferroptosis inhibition, but its efficacy in OA remains to be clarified.^[^ ^18^, ^19^ ^]^ Previous studies have indicated that the therapeutic effect of DEF alone is limited.^[^ ^3^ ^]^ Although DEF can reduce iron overload, it can also form redox‐active complexes, interfering with the Fenton reaction and shifting the cellular redox balance toward ROS generation, which is detrimental to ferroptosis inhibition.^[^ ^20^, ^21^ ^]^ Recently, cerium (Ce)‐containing nanoparticles have emerged as antioxidants due to their superoxide dismutase, catalase, and peroxidase activities.^[^ ^22^, ^23^ ^]^ Ce‐containing nanoparticles exhibit excellent ROS‐scavenging activity and have been widely used in ROS‐related diseases. For example, in cancer, Alzheimer's disease, inflammatory liver diseases, and acute kidney injury, cerium nanoparticles demonstrate antioxidant activity by scavenging various free radicals, showing substantial potential in ferroptosis inhibition.^[^ ^24^, ^25^, ^26^ ^]^ Therefore, incorporating DEF into Ce‐containing nanoparticles might counteract DEF‐induced ROS elevation, resulting in enhanced effects on ferroptosis inhibition.

Moreover, repairing damaged cartilage matrix while inhibiting chondrocyte ferroptosis is crucial. Pterostilbene (PTE), a resveratrol analog found in blueberries, possesses antioxidant, antitumor, and anti‐inflammatory activities, inhibiting cellular inflammation, oxidative stress, apoptosis, and ferroptosis.^[^ ^27^, ^28^ ^]^ Recent studies have confirmed PTE's ability to reduce chondrocyte loss and promote ECM repair in OA‐damaged chondrocytes.^[^ ^29^ ^]^ PTE may hold therapeutic potential in repairing damaged cartilage.

In this study, we developed a drug delivery method for Ce@D&P NPs, designed to target iron ions and ROS through multitarget regulation, effectively inhibiting chondrocyte ferroptosis and promoting cartilage ECM repair, thereby achieving improved therapeutic efficacy for OA. We successfully prepared and characterized Ce@D&P NPs. IL‐1β‐stimulated chondrocytes and a destabilization of the medial meniscus (DMM) mouse OA model were used to verify the therapeutic effects of Ce@D&P NPs. We also performed transcriptomics and metabolomics analyses to discuss the potential mechanisms of Ce@D&P NPs. Overall, this study developed a novel multitarget therapeutic nanoparticle delivered via intra‐articular injection, providing a promising strategy for OA clinical treatment.

## 2 Results

### 2.1 Inflammation‐Driven GPX4 Downregulation Promotes Ferroptosis in Osteoarthritic Cartilage

**Figure 1:**
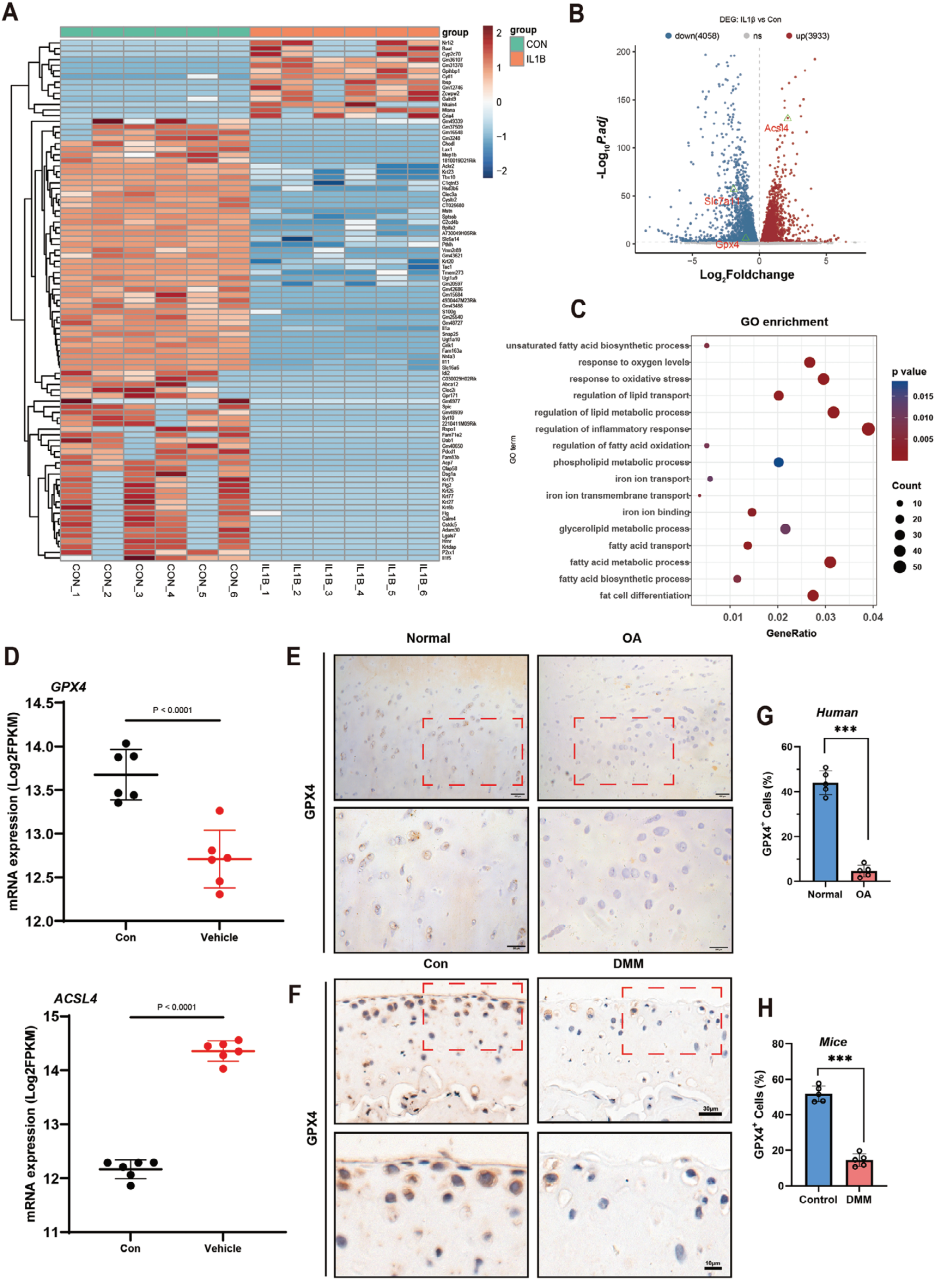
Inflammation‐Driven GPX4 downregulation promotes ferroptosis in osteoarthritic cartilage. A) Heatmap of DEGs in normal chondrocytes and IL‐1β‐stimulated chondrocytes (*n* = 6). B) Volcano plot of DEGs between IL‐1β‐stimulated and normal chondrocytes. C) GO enrichment analysis of DEGs between the two groups. D) mRNA expression levels of *Gpx4* and *Acsl4* in normal and IL‐1β‐stimulated chondrocytes. E) Immunohistochemical staining of GPX4 in normal cartilage and cartilage from OA patients. F) Immunohistochemical staining of GPX4 in cartilage from normal mice and DMM model mice. G) Quantitative analysis of GPX4 expression in human cartilage (*n* = 5). H) Quantitative analysis of GPX4 expression in mouse cartilage (*n* = 5). (*: *p* < 0.05, **: *p* < 0.01, ***: *p* < 0.001).

To elucidate the potential causes of cartilage degeneration, chondrocytes were stimulated with IL‐1β for 24 h, and RNA‐seq analysis was performed on IL‐1β‐stimulated and normal chondrocytes. Differentially expressed genes (DEGs) were defined with a p.adjust < 0.05 and |log2 FC| > 1. The results identified a total of 1622 DEGs, with 574 upregulated and 1048 downregulated genes (**Figure** **1**A,B). Principal component analysis (PCA) showed significant differences in mRNA expression levels between IL‐1β‐stimulated degenerative chondrocytes and normal chondrocytes (Figure S1B, Supporting Information). Gene Ontology (GO) enrichment analysis using the DAVID database indicated that the DEGs between the two groups were associated with biological processes, such as response to oxidative stress, iron ion transport, and iron ion binding (Figure 1C). Additionally, transcriptome data suggested that IL‐1β stimulation reduced the expression of glutathione peroxidase *GPX4* and increased the expression of long‐chain acyl‐CoA synthetase 4 (*ACSL4*) (Figure 1E). Gene set enrichment analysis (GSEA) showed that IL‐1β intervention upregulated genes related to Inflammatory Response LPS Up and Iron Import Into Cell in chondrocytes (Figure S1C, Supporting Information). Immunohistochemical staining indicated decreased GPX4 expression in cartilage tissue from OA patients and DMM model mice (Figure 1E–H). These results suggest that inflammation‐induced OA cartilage degeneration is associated with ferroptosis.

### 2.2 Preparation and Characterization of Ce@D&P NPs

**Figure 2:**
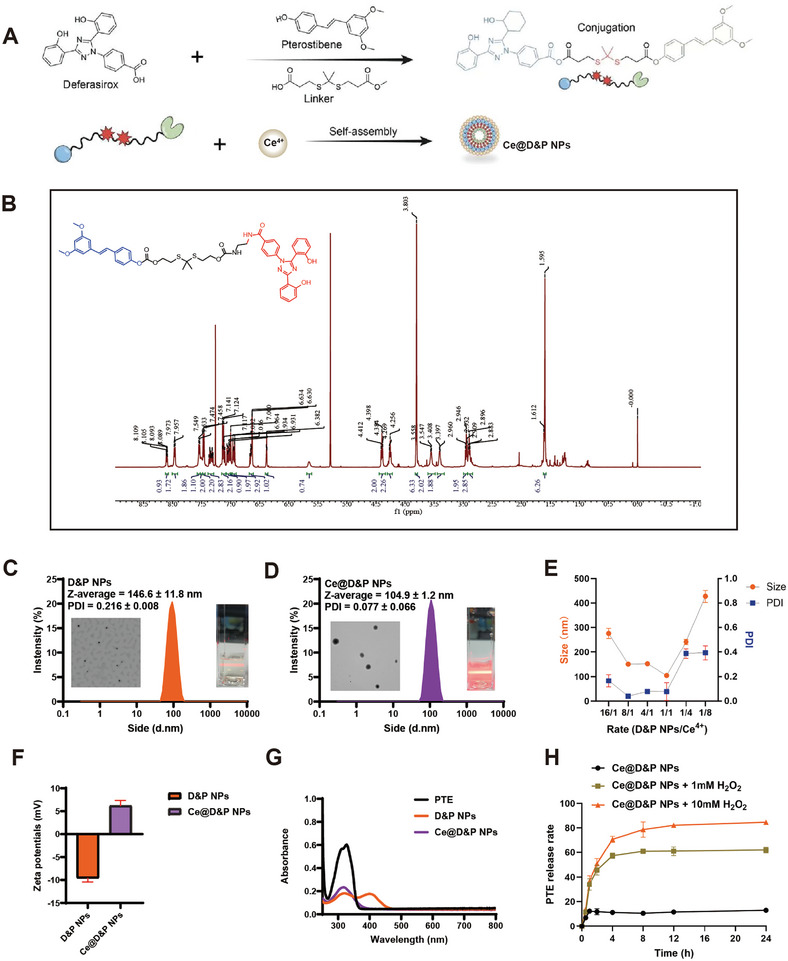
Preparation and Characterization of Ce@D&P NPs. A) Schematic diagram of the preparation of Ce@D&P NPs. B) ^1^H NMR spectrum of Ce@D&P NPs. C,D) Particle size, representative transmission electron microscopy (TEM) images, and Tyndall effect images of D&P NPs and Ce@D&P NPs. E) Average particle size and polydispersity index (PDI) of D&P NPs combined with Ce^4+^ in different ratios. F) Zeta potential of D&P NPs and Ce@D&P NPs. G) UV–vis spectra of PTE, D&P NPs, and Ce@D&P NPs. H) Release profile of Ce@D&P NPs under stimulation with 1 mm H_2_O_2_ and 10 mm H_2_O_2_.

According to the literature method,^[^ ^30^ ^]^ a thioketal linker was synthesized, with DEF and PTE molecules bonded on either side to form D&P nanoparticles. Mass spectrometry and ^1^H NMR spectra confirmed the successful preparation of the nanoparticles (**Figure** **2**A,B; and Figures S2–S5, Supporting Information). These were then coassembled with CeCl_3_ via a precipitation method to prepare Ce@D&P NPs (Figure 2A).

The particle size and zeta potential of D&P NPs were measured using a Malvern particle size analyzer. The results showed that the particle size of D&P NPs was around 100 nm, with a Z‐Average of 146.6 ± 11.5 nm and a polydispersity index (PDI) of 0.216 ± 0.008. Transmission electron microscopy (TEM) images showed spherical and smooth particles (Figure 2C,D). To further optimize the ratio, gradient grouping of D&P NPs and Ce was performed during the preparation of Ce@D&P NPs, and their particle sizes and PDI were measured. The optimal particle size (104.9 nm) and PDI (0.077) were obtained under the 1:1 condition (Figure 2E). The zeta potential results indicated a reversal in the potential of D&P after loading with Ce^4+^ (from −9.59 to 6.20 mV), providing strong evidence for the successful construction of Ce@D&P NPs (Figure 2F). Additionally, the UV–vis spectra show the absorption profiles of PTE, D&P NPs, and Ce@D&P NPs. The spectrum of PTE has an obvious absorption peak around 300 nm. In contrast, the D&P NPs prepared from DEF and PTE have a newly emerged absorption peak at around 400 nm. As for the Ce@D&P NPs composed of Ce and D&P NPs, it also has an absorption peak at around 300 nm. However, compared with the corresponding absorption peak of PTE, the intensity of this absorption peak has decreased (Figure 2G). The drug release profile of Ce@D&P NPs in different concentrations of H_2_O_2_ was determined by high‐performance liquid chromatography (HPLC). The results showed that drug release increased with the concentration of H_2_O_2_. Under 1 mm H_2_O_2_ stimulation, Ce@D&P NPs released ≈61% of PTE within 12 h, while under 10 mm H_2_O_2_, Ce@D&P NPs released about 82.13% of PTE within 12 h. In contrast, only 13.07% of the drug was released in the phosphate buffer saline (PBS) control group after 24 h. This indicates that higher ROS concentrations may lead to higher PTE release (Figure 2H).

In addition, we evaluated the effects of Ce@D&P NPs at different concentrations and their respective components (PTE, DEF, CeCl_3_) on the cell viability of chondrocytes. The drug concentrations we selected were all within the safe range (Figure S6A–D, Supporting Information). And we performed HE staining of the viscera of mice after intervention with Ce@N&P NPs. The results showed that there is no significant difference in the tissue morphology of the heart, lung, spleen, liver, and kidney between the Ce@D&P NPs‐treated group and the control group. This indicates that Ce@D&P NPs do not cause noticeable tissue damage or pathological changes in these organs, indicating their safety (Figure S6E, Supporting Information).

### 2.3 Ce@D&P NPs Inhibit IL‐1β‐Induced Ferroptosis in Chondrocyte

**Figure 3:**
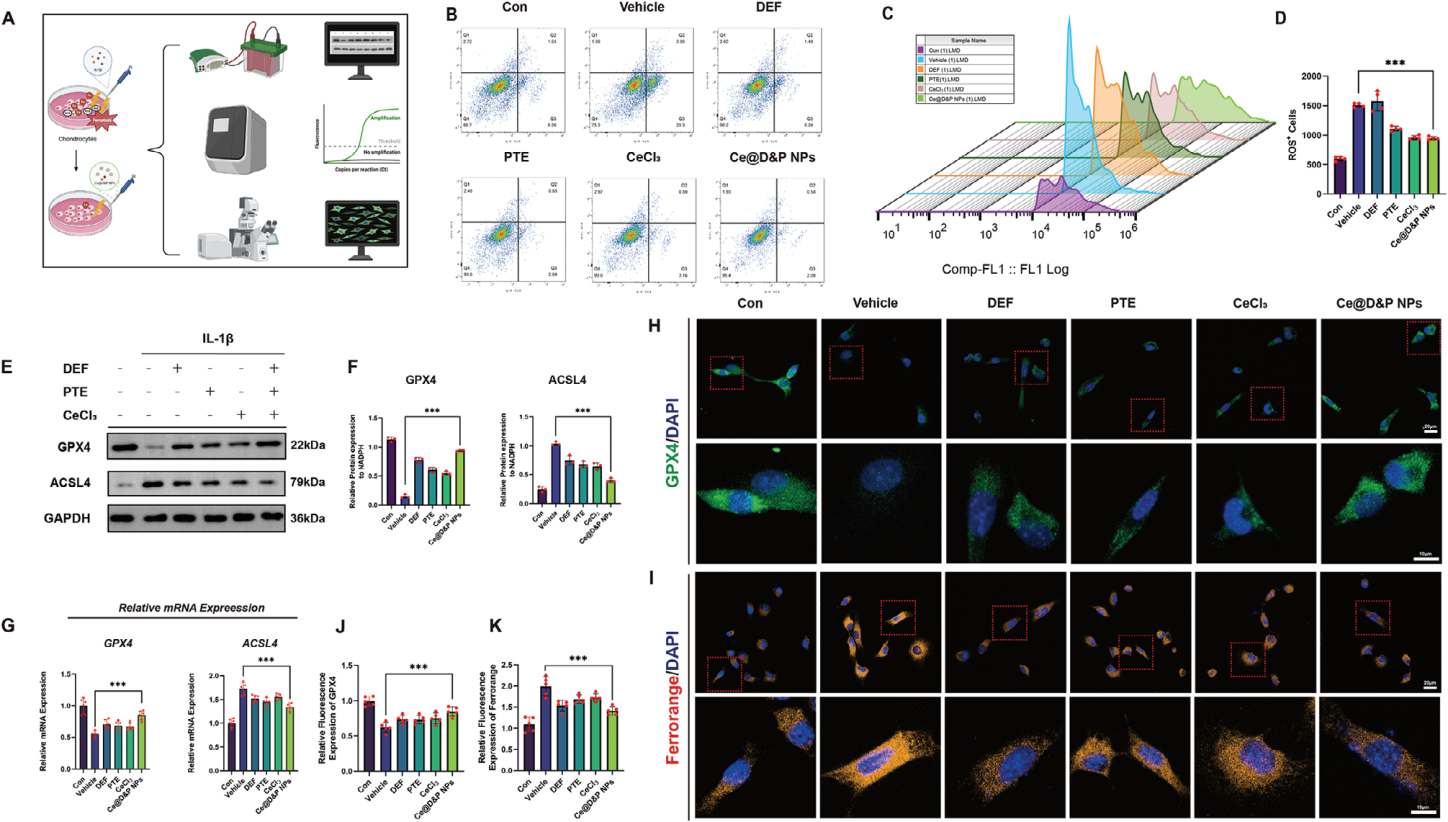
Ce@D&P NPs Inhibit IL‐1β‐Induced Ferroptosis in Chondrocyte. A) Construction of a chondrocyte ferroptosis model using IL‐1β stimulation. B) Evaluation of chondrocyte apoptosis by flow cytometry using Annexin V‐FITC and PI staining. C,D). Detection and quantification of ROS‐positive cells in chondrocytes by flow cytometry. E) Western blot bands of GPX4 and ACSL4 under different intervention conditions after IL‐1β stimulation. F) Relative protein expression levels of GPX4 and ACSL4. G) Relative mRNA expression levels of *GPX4* and *ACSL4* under different intervention conditions after IL‐1β stimulation. H) Immunofluorescence staining of GPX4 in chondrocytes under different interventions. I) Immunofluorescence staining of FerroOrange in chondrocytes under different interventions. J) Quantitative analysis of GPX4 fluorescence intensity in each group. K) Quantitative analysis of FerroOrange fluorescence intensity in each group. Values and error bars represent mean ± standard deviation (*n* ≥ 3). (*: *p* < 0.05, **: *p* < 0.01, ***: *p* < 0.001).

To elucidate the role of Ce@D&P NPs in inhibiting ferroptosis, a chondrocyte ferroptosis model was constructed by stimulating chondrocytes with IL‐1β (**Figure** **3**A). First, we observed chondrocyte apoptosis under different interventions using flow cytometry, and the results indicated that Ce@D&P NPs could effectively alleviate IL‐1β‐induced chondrocyte apoptosis (Figure 3B). Additionally, we evaluated ROS levels in chondrocytes of each group using an ROS detection kit. Flow cytometry and confocal microscopy results showed that IL‐1β stimulation significantly increased ROS levels in chondrocytes, while Ce@D&P NPs treatment reduced ROS levels (Figure 3C; and Figure S7A,B, Supporting Information). TEM images demonstrated that in the chondrocytes of the Vehicle group treated with IL‐1β, mitochondria exhibited the characteristics of ferroptosis, such as a reduction in mitochondrial size and a decrease or disappearance of mitochondrial cristae. However, intervention with Ce@D&P NPs could reverse this phenomenon and improve the mitochondrial morphology in chondrocytes (Figure S8A, Supporting Information). Western blot (WB) results demonstrated that IL‐1β intervention led to a decrease in GPX4 protein expression levels, while the protein levels of ACSL4 increased. Treatment with Ce@D&P NPs effectively increased GPX4 protein expression levels and decreased ACSL4 protein levels (Figure 3E,F). Furthermore, we examined ferroptosis‐related marker genes at the genetic level using qPCR. The results indicated that compared to the control group, Ce@D&P NPs treatment increased *GPX4* mRNA levels and decreased *ACSL4* mRNA levels (Figure 3G). Immunofluorescence images and quantitative results also showed that Ce@D&P NPs could reverse the reduction of GPX4 induced by IL‐1β intervention (Figure 3H,J). Malondialdehyde (MDA) is one of the end products of lipid peroxidation. The MDA levels in each group were evaluated using the MDA detection kit (Beyotime, S0131S). The results showed that the MDA level was elevated in the chondrocytes of the Vehicle group treated with IL‐1β, and after intervention with Ce@D&P NPs, the MDA level in chondrocytes was significantly decreased. These additional results will provide further evidence of the involvement of ferroptosis and demonstrate that the drug can inhibit ferroptosis in chondrocytes (Figure S8B, Supporting Information). Meanwhile, the expression of GSH was decreased in chondrocytes stimulated by IL‐1β, and the GSH level was significantly restored after intervention with Ce@D&P NPs (Figure S8C, Supporting Information). Considering that Fe ions are crucial indicators of oxidative stress, we stained ferrous ions in chondrocytes of each group using FerroOrange. The results showed that DEF, D&P NPs, and Ce@D&P NPs all exhibited inhibitory effects on iron ions, with Ce@D&P NPs showing superior inhibitory effects (Figure 3I,K). We also detected the iron metabolism‐related protein (Ferritin) by WB. Combining with the quantification results, it was found that the content of Ferrtin was decreased in the Vehicle group, while the relative protein expression of Ferrtin was increased in the Ce@D&P NPs group (Figure S8D,E, Supporting Information). These results suggest that Ce@D&P NPs can effectively inhibit IL‐1β‐induced chondrocyte ferroptosis.

### 2.4 Transcriptomic and Metabolomic Shifts Following Ce@D&P NPs Intervention in IL‐1β‐Treated Chondrocytes

**Figure 4:**
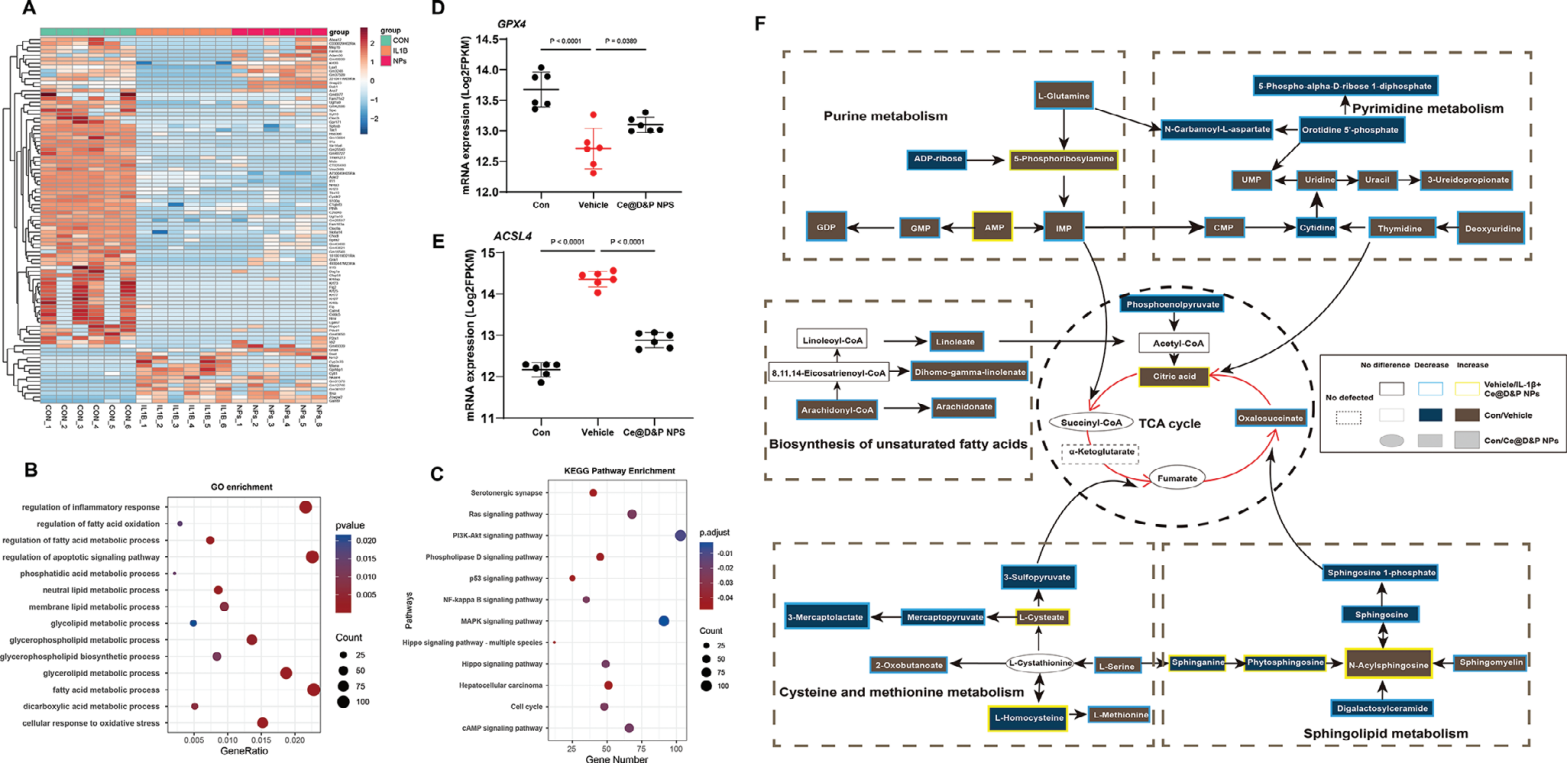
Transcriptomic and Metabolomic Shifts Following Ce@D&P NPs Intervention in IL‐1β‐Treated Chondrocytes. A) Heatmap of differentially expressed genes (DEGs) between Ce@D&P NPs+IL‐1β, IL‐1β‐stimulated vehicle group, and control group (*n* = 6). B) GO enrichment analysis of DEGs between Ce@D&P NPs+IL‐1β and IL‐1β‐treated vehicle group. C) KEGG pathway enrichment analysis of DEGs between Ce@D&P NPs+IL‐1β and IL‐1β‐treated vehicle group. D,E) Expression levels of *ACSL4* and *GPX4* between Ce@D&P NPs+IL‐1β and IL‐1β‐treated vehicle group. F) Metabolomics network analysis of Ce@D&P NPs+IL‐1β, vehicle group, and normal group. (Yellow, blue, and black rectangular borders represent increased, decreased, and unchanged differential metabolites in Ce@D&P NPs+IL‐1β compared to the vehicle group, respectively; brown, blue, and white filled rectangles represent increased, decreased, and unchanged differential metabolites in the vehicle group compared to the control group, respectively; large gray rectangles, small gray rectangles, and gray ellipses represent increased, decreased, and unchanged differential metabolites in Ce@D&P NPs+IL‐1β compared to the control group, respectively; dashed rectangles indicate undetected metabolites).

In the IL‐1β group and Ce@D&P NPs group, a total of 32 577 genes were identified, among which 6083 genes showed differential expression (|log2| fold change > 1, *p* < 0.05) (**Figure** **4**A; and Figure S9A,B, Supporting Information). The gene expression patterns of the three groups displayed distinct characteristics in PCA, consistent with RNA‐seq results (Figure S9C, Supporting Information). GO enrichment analysis revealed that DEGs after Ce@D&P NPs intervention were mainly associated with lipid metabolism processes such as regulation of fatty acid oxidation, neutral lipid metabolic process, and glycolipid metabolic process (Figure 4B). KEGG pathway enrichment analysis using the clusterProfiler package indicated that Ce@D&P NPs intervention might exert effects through pathways, such as the PI3K‐Akt signaling pathway, NF‐kappa B signaling pathway, and MAPK signaling pathway (Figure 4C). Additionally, Ce@D&P NPs increased the expression of *GPX4* and inhibited the expression of *ACSL4* (Figure 4D,E). GSEA was employed to determine the impact of Ce@D&P NPs treatment on various cellular processes. Our findings indicate that compared to the IL‐1β group, Ce@D&P NPs treatment inhibited the expression of genes related to pathways such as Iron Ion Homeostasis, Oxidative Stress and Redox Pathway, Inflammatory Response, Lipid Oxidation, Peroxisomal Lipid Metabolism, and Mitochondrial Fatty Acid Beta Oxidation (Figure S9D, Supporting Information).

To further investigate the mechanisms of Ce@D&P NPs, we conducted metabolomics analysis on CDC. Multivariate analysis of the metabolic profile data from the Con group, IL‐1β group, and IL‐1β + Ce@D&P NPs group was performed using partial least squares discriminant analysis (PLS‐DA). PCA results indicated spatial differences in the differential metabolites between the Con group, IL‐1β group, and IL‐1β + Ce@D&P NPs group (Figure S10B, Supporting Information). Compared to the IL‐1β group, the Ce@D&P NPs treatment group exhibited significant metabolic reprogramming, including metabolites, such as Xenognosin A, Venlafaxine, Suillusin, and Spirapril (Figure S10A, Supporting Information). Based on the metabolomics analysis, a metabolic map of chondrocytes under different interventions was established (Figure 4I). We found that Ce@D&P NPs could reverse the changes in normal metabolites induced by IL‐1β stimulation through various metabolic pathways, including a) Purine metabolism; b) Pyrimidine metabolism; c) Biosynthesis of unsaturated fatty acids; d) Cysteine and methionine metabolism; e) Sphingolipid metabolism; f) TCA cycle (Figure S9E, Supporting Information).

For the metabolomic sequencing results, we further analyzed the relationship between differential genes and differential metabolites in the IL‐1β group and the IL‐1β+Ce@D&P NPs group. The results of the correlation network diagram showed that there was a strong negative correlation between the metabolite Docosahexaenoic acid and the ferroptosis‐related gene *Gpx4*. There was a strong positive correlation between L‐Glutamic acid and the ferroptosis‐related gene solute carrier family 7 member 11 (*Slc7a11*) (Figure S10C, Supporting Information). The results of these omics studies have provided valuable directions for the molecular biological mechanisms by which nanomaterials inhibit ferroptosis of chondrocytes under disease conditions.

### 2.5 Ce@D&P NPs Inhibit IL‐1β‐Induced ECM Damage in Chondrocytes

**Figure 5:**
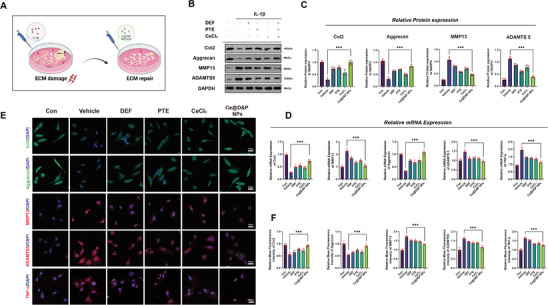
Ce@D&P NPs Inhibit IL‐1β‐Induced ECM Damage in Chondrocytes. A) Schematic diagram of the chondrocyte ECM damage model using IL‐1β. B) Western blot bands of Col2, Aggrecan, MMP13, and ADAMTS5 under different intervention conditions after IL‐1β stimulation. C) Relative protein expression levels of Col2, Aggrecan, MMP13, and ADAMTS5. D) Relative mRNA expression levels of *Col2, Aggrecan, MMP13, ADAMTS5*, and *TNF‐α* under different intervention conditions after IL‐1β stimulation. E) Immunofluorescence staining of Col2, Aggrecan, MMP13, ADAMTS5, and TNF‐α in chondrocytes under different interventions. F) Quantitative analysis of fluorescence intensity for Col2, Aggrecan, MMP13, ADAMTS5, and TNF‐α. Values and error bars represent mean ± standard deviation (*n* ≥ 3). (*: *p* < 0.05, **: *p* < 0.01, ***: *p* < 0.001).

We used IL‐1β to establish a chondrocyte ECM damage model to further investigate the effects of Ce@D&P NPs on chondrocyte ferroptosis and ECM (**Figure** **5**A). The expression levels of ECM components in CDC treated and untreated with Ce@D&P NPs were compared using RT‐qPCR and WB analysis. WB results indicated that IL‐1β stimulation led to a decrease in Col2 and Aggrecan protein levels and an increase in MMP13 and ADAMTS5 protein levels. Ce@D&P NPs effectively increased the protein expression of Col2 and Aggrecan while inhibiting the expression of MMP13 and ADAMTS5 (Figure 5B,C). Similarly, qPCR results showed the same trend as WB, with Ce@D&P NPs intervention increasing the mRNA expression of ECM anabolic markers (*Col2* and *Aggrecan*) and decreasing the expression of catabolic markers (*MMP13* and *ADAMTS5*). TNF‐α is a key proinflammatory cytokine that plays an important role in the inflammatory response. Elevated levels of TNF‐α in osteoarthritis can have profound effects on chondrocyte function and ECM synthesis and degradation.^[^ ^31^ ^]^ To verify the potential of PTE and Ce@D&P NPs in anti‐inflammatory regulation of ECM, we also detected the expression of TNF‐α in chondrocytes of each group. The qPCR results indicated that Ce@D&P NPs containing PTE can reduce the expression of the inflammatory factor *TNF‐α*. (Figure 5D). Immunofluorescence and quantitative results suggested that Ce@D&P NPs enhanced the expression of Col2 and Aggrecan while inhibiting MMP13, ADAMTS5, and TNF‐α expression (Figure 5E,F).

### 2.6 Ce@D&P NPs Alleviate Inflammation and Ferroptosis in DMM‐Induced Mouse Cartilage

**Figure 6:**
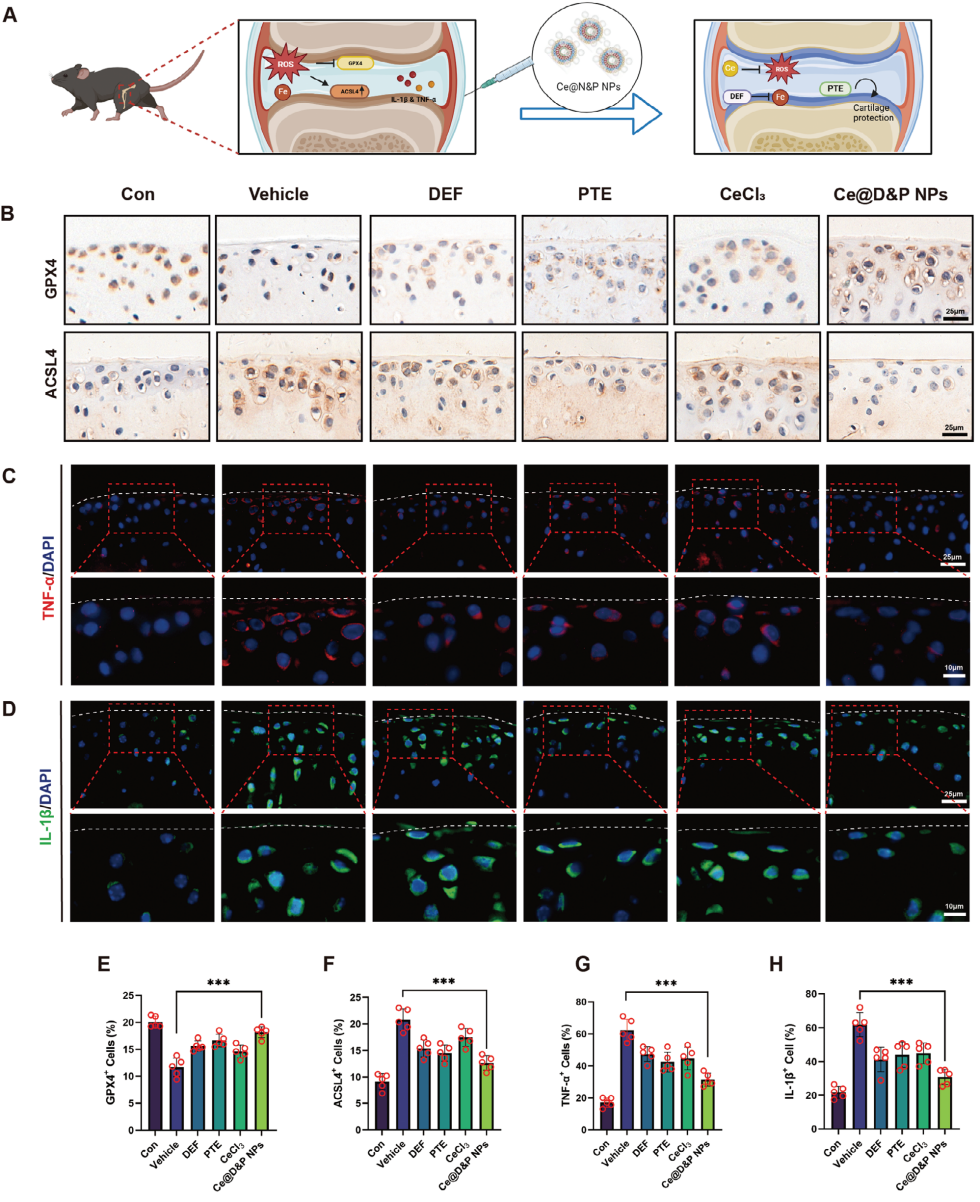
Ce@D&P NPs Alleviate Inflammation and Ferroptosis in DMM‐Induced Mouse Cartilage. A) Schematic diagram of Ce@D&P NPs alleviating cartilage damage in OA model mice. B) IHC images of GPX4 and ACSL4. C,D) Immunofluorescence images of TNF‐α and IL‐1β. E) Quantification of GPX4‐positive cells in each group. F) Quantification of ACSL4‐positive cells in each group. G) Quantification of TNF‐α fluorescence intensity in each group. H) Quantification of IL‐1β fluorescence intensity in each group. Values and error bars represent mean ± standard deviation (*n* ≥ 5). (*: *p* < 0.05, **: *p* < 0.01, ***: *p* < 0.001).

To investigate the in vivo anti‐inflammatory and antiferroptotic efficacy of Ce@D&P NPs, we employed the DMM model to induce OA in mice. Immunohistochemistry (IHC) images and quantitative results showed that interventions with Ce@D&P NPs effectively increased the expression of GPX4 and inhibited the expression of ACSL4 (**Figure** **6**B,E,F). Additionally, immunofluorescence results indicated that Ce@D&P NPs reduced the levels of inflammatory factors IL‐1β and TNF‐α in the cartilage of OA mice (Figure 6C,D,G,H). These results suggest that Ce@D&P NPs can alleviate inflammation and ferroptosis in the OA mouse model.

### 2.7 Ce@D&P NPs Alleviate DMM‐Induced OA in Mice

**Figure 7:**
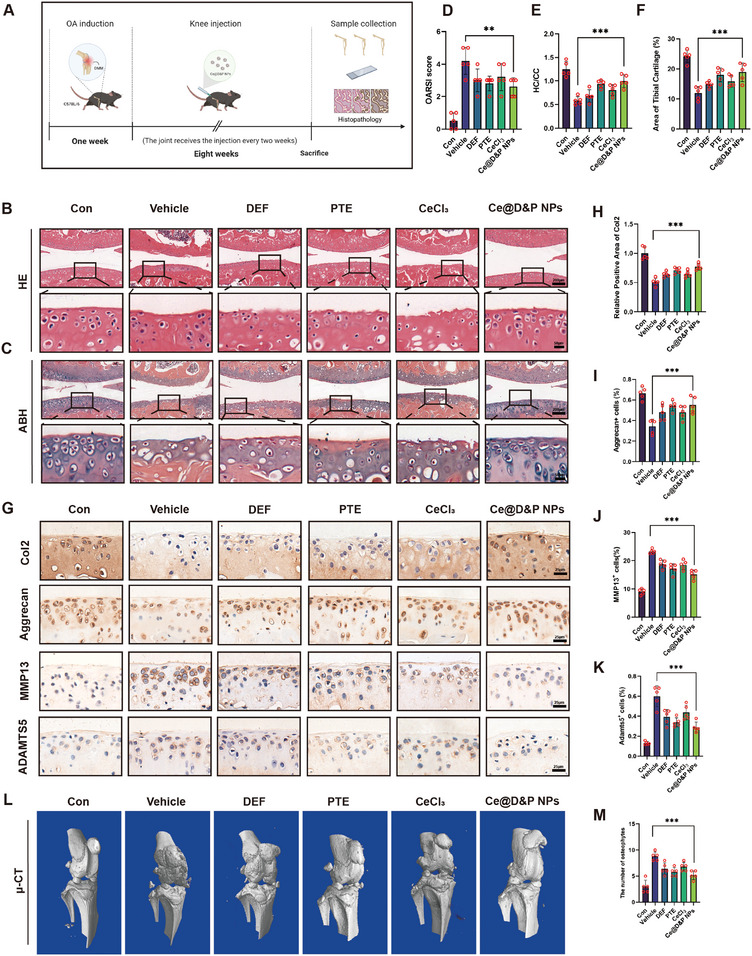
Ce@D&P NPs Alleviate DMM‐Induced OA in Mice. A) Schematic diagram of the experimental design with DMM‐induced OA mouse model and intra‐articular injection of treatments. B,C) HE staining and ABH staining of knee joint sections from each group. D) OARSI scores indicating the degree of knee joint degeneration in mice. E) Quantification of the ratio of hyaline cartilage (HC) versus calcified cartilage (CC). F) Quantification of the area tibial cartilage. G) Representative IHC images of Col2, Aggrecan, MMP13, ADAMTS5 expression in knee cartilage. H–K) Quantification of Col2 and Aggrecan, MMP13, ADAMTS5 cells in knee cartilage from different groups. L) Micro‐CT images of knee joints from different groups. M) Quantification of osteophyte formation in knee joints from different groups. Values and error bars represent mean ± standard deviation (*n* ≥ 5). (*: *p* < 0.05, **: *p* < 0.01, ***: *p* < 0.001).

To gain deeper insights into the positive impact of Ce@D&P NPs on cartilage matrix restoration, we utilized a DMM‐induced OA mouse model and administered Ce@D&P NPs via intra‐articular injection at a volume of 8 µL every 2 weeks. Mice were sacrificed, and knee joint samples were collected after 8 weeks (**Figure** **7**A). Hematoxylin and eosin (HE) and Alcian Blue Hematoxylin (ABH) staining results showed significant cartilage erosion in the knee joints of OA mice, with higher OARSI scores indicating severe degeneration in the vehicle model group. In contrast, the Ce@D&P NPs group exhibited lower OARSI scores and intact cartilage matrix compared to the vehicle group (Figure 7B–D). Meanwhile, the Ce@D&P NPs group was able to reverse the reduction in the areas of the tibial cartilage and HC/CC in the modeled mice (Figure 7E,F). IHC results indicated significant cartilage matrix damage in the DMM model, accompanied by a loss of Col2, Aggrecan and increased expression of MMP13, ADAMTS5. However, Ce@D&P NPs effectively increased the expression of Col2 and Aggrecan, the markers of cartilage matrix synthesis, and inhibited the expression of MMP13 and ADAMTS5, the markers of cartilage matrix degradation (Figure 7G–K). 3D reconstructed images from micro‐CT showed rough knee joint surfaces, prominent osteophyte formation, and severe joint damage in the DMM group. Treatment with Ce@D&P NPs significantly reduced osteophyte formation and alleviated joint damage (Figure 7L,M). These results indicate that Ce@D&P NPs can effectively mitigate cartilage damage in OA mice.

## 3 Discussion

Osteoarthritis (OA) is the most prevalent joint disease, with an increasing trend. Recent studies have emphasized the role of ferroptosis in the pathogenesis of OA, highlighting the potential of targeting chondrocyte ferroptosis to slow OA progression.^[^ ^32^, ^33^ ^]^ In this study, we designed and constructed Ce@D&P NPs. On one hand, Ce@D&P NPs target ferroptosis by using deferasirox (DEF) and cerium (Ce) to reduce ROS and Fe levels, thus protecting chondrocytes from ferroptosis. On the other hand, Ce@D&P NPs aim to directly repair the extracellular matrix (ECM) through pterostilbene (PTE), correcting the metabolic imbalance of chondrocyte ECM. The multifunctionality of Ce@D&P NPs and their good biocompatibility make them a promising candidate for the treatment of OA.

We validated the intermediates and final Ce@D&P NPs using nuclear magnetic resonance (NMR) and mass spectrometry. Previous studies have shown that smaller NP sizes facilitate cellular uptake.^[^ ^34^ ^]^ Therefore, we explored different ratios of PTE and DEF and ultimately achieved an optimal polydispersity index (PDI) and an acceptable nanoparticle size (146 nm) with a 1:1 ratio, enabling efficient cellular uptake of Ce@D&P NPs. The rounded spherical structure of Ce@D&P NPs helps avoid mechanical damage to cells. Considering the avascular nature of cartilage, we enhance cartilage repair through intra‐articular injection. Previous studies have also indicated that intra‐articular injection is a more effective delivery strategy compared to oral or intravenous administration.^[^ ^35^, ^36^, ^37^ ^]^ Additionally, to achieve the self‐decomposition of Ce@D&P NPs and timely release of their contents, we simulated the in vivo ROS environment using H_2_O_2_. We found that the release of Ce@D&P NPs increased with the concentration of ROS, consistent with previous reports.^[^ ^38^, ^39^ ^]^

To evaluate the effectiveness of Ce@D&P NPs in achieving multitarget regulation, we constructed a chondrocyte ferroptosis model using IL‐1β. The results showed that after IL‐1β treatment, the levels of ROS and ACSL4 in chondrocytes increased, while GPX4 levels decreased. This is consistent with previous research, indicating increased chondrocyte ferroptosis in an in vitro inflammatory environment.^[^ ^40^ ^]^ Additionally, 2’,7’‐dichlorodihydrofluorescein diacetate (DCFH‐DA) detection revealed that ROS levels were significantly reduced in groups containing Ce, as reported in previous literature.^[^ ^41^, ^42^ ^]^ Recent studies also suggest that the valence state conversion between Ce^3+^ and Ce^4+^ on the surface of cerium ions provides ROS‐scavenging properties, and nanoceria can interact with ROS, reducing superoxide radicals or oxidizing hydrogen peroxide.^[^ ^43^ ^]^ Furthermore, we found that intervention with DEF alone increased ROS levels. This might be due to the chelator sequestering some iron from transferrin, disrupting the original redox balance.^[^ ^20^ ^]^ In contrast, ROS expression in the Ce@D&P NPs groups remained significantly lower than in the IL‐1β group, indicating that the nanoparticles still exhibited anti‐ROS properties. This could be because the ROS‐reducing effect of Ce is more pronounced than the ROS‐increasing effect of DEF, and PTE itself may also exhibit some antioxidant effects.^[^ ^44^ ^]^ We also used FerroOrange to detect iron content in chondrocytes under different interventions. The results indicated that iron ion expression was effectively reduced in all experimental groups containing DEF. This confirms that the iron‐chelating biological function of DEF is retained in the Ce@D&P NPs formulation.^[^ ^45^ ^]^ By examining ferroptosis markers, such as ACSL4, GPX4, and P53, our data suggest that ferroptosis occurs in OA chondrocytes and that Ce@D&P NPs can effectively inhibit ferroptosis.^[^ ^46^, ^47^ ^]^

Recent studies have shown that cellular lipids and lipid metabolism play a crucial role in ferroptosis, with levels of peroxidized polyunsaturated lipids and related lipid metabolic enzymes determining ferroptosis sensitivity.^[^ ^48^ ^]^ Highly oxidizable polyunsaturated fatty acids (PUFAs) can copropagate lipid peroxidation with phospholipids, exacerbating ferroptosis, which is central to the execution of ferroptosis.^[^ ^49^ ^]^ Our transcriptomics analysis also yielded results related to lipid metabolism, indicating that Ce@D&P NPs intervention can regulate biological processes, such as the regulation of fatty acid oxidation, neutral lipid metabolic process, and glycolipid metabolic process. Additionally, we discovered several signaling pathways that communicate between lipid metabolism and ferroptosis, such as the PI3K‐Akt signaling pathway and MAPK signaling pathway. Previous studies have indicated that lipogenesis is an essential function of the PI3K‐AKT‐mTOR axis, with mTOR1 promoting GPX4 synthesis to regulate ferroptosis.^[^ ^50^, ^51^ ^]^ Activation of AMPK inhibits fatty acid synthesis and increases fatty acid oxidation, playing a role in inhibiting ferroptosis.^[^ ^52^, ^53^ ^]^ In summary, Ce@D&P NPs are likely to inhibit chondrocyte ferroptosis by regulating the transcription of ferroptosis‐related genes and reshaping lipid metabolism in chondrocytes. To gain a more comprehensive understanding of the potential mechanisms by which Ce@D&P NPs regulate ferroptosis, we also conducted metabolomics analysis. The results revealed that Ce@D&P NPs are involved in regulating not only lipid metabolism‐related sphingolipid metabolism but also cysteine and methionine metabolism pathways. Previous research has confirmed that inhibiting sphingolipid synthesis can reduce ferroptosis in HT22 cells by stimulating the HIF‐1 pathway.^[^ ^54^ ^]^ This is consistent with our metabolomics results, showing a reduction in sphingolipid metabolites in the Ce@D&P NPs intervention group. Moreover, we found that Ce@D&P NPs can increase the expression of cysteine metabolism products. It is well known that glutathione (GSH) is a critical endogenous antioxidant and a key regulator of ferroptosis.^[^ ^55^ ^]^ Cysteine is an essential component of GSH, and methionine can be converted into cysteine through a series of metabolic pathways.^[^ ^56^ ^]^ Studies have shown that enhancing cysteine uptake can effectively counteract excess ROS produced by the Fenton reaction. The increase in cysteine expression by Ce@D&P NPs may be one of the potential mechanisms by which they exert their effects.^[^ ^57^ ^]^ Therefore, our multiomics analysis results suggest that the regulation of ferroptosis by Ce@D&P NPs is complex, involving transcriptional regulation of lipid metabolism‐related genes and metabolic regulation of cysteine metabolism and other energy metabolism processes. This is similar to the findings of a recent clinical metabolomics study on OA.^[^ ^58^ ^]^ Further exploration is needed in the future to fully elucidate these mechanisms.

Previous studies have shown that iron overload in chondrocytes leads to increased intracellular ROS levels and downregulation of GPX4. This not only enhances the susceptibility of chondrocytes to oxidative stress but also stimulates the secretion of inflammatory mediators, such as IL‐1β and matrix metalloproteinases (MMP13), exacerbating ECM degradation, and accelerating OA progression.^[^ ^12^, ^59^ ^]^ Moreover, research indicates that the ferroptosis inducer erastin can accelerate cartilage degradation, while the ferroptosis inhibitor ferrostatin‐1 (fer‐1) can reduce IL‐1β‐induced lipid peroxidation and ferroptosis, thereby slowing cartilage degradation.^[^ ^6^ ^]^ This is consistent with our findings, as we observed that Ce@D&P NPs can also inhibit ferroptosis (increase GPX4 and decrease ACSL4) and promote cartilage ECM synthesis while inhibiting ECM degradation, thus demonstrating a cartilage repair effect. As previously mentioned, Ce and DEF in Ce@D&P NPs inhibit chondrocyte ferroptosis by eliminating ROS and reducing iron load. A recent study found that GPX4 not only inhibits ferroptosis but also prevents ECM degradation.^[^ ^59^ ^]^ Therefore, Ce@D&P NPs may be able to prevent the further progression of OA by inhibiting chondrocyte ferroptosis. Recent research has shown that PTE reduces NLRP3 inflammatory activation and the release of inflammatory cytokines by inactivating NF‐κB signaling, and it can upregulate COL2 while downregulating MMP13 and ADAMTS5 expression to prevent chondrocyte degradation.^[^ ^29^, ^60^ ^]^ In this study, we also found that Ce@D&P NPs can inhibit the inflammatory factor TNF‐α and promote cartilage anabolic metabolism while inhibiting catabolic metabolism. Thus, the application of PTE enables Ce@D&P NPs to balance cartilage matrix metabolism and promote cartilage regeneration.

Our study has several limitations. Our experiments were conducted only on cell cultures and mice, without including large animal samples, thus remaining at the preclinical research stage. Clinical research is insufficient. In future studies, we need to increase the sample size and include large animal models. Additionally, we need to focus on the duration of the antioxidant effects of Ce@D&P NPs, to prevent an imbalance between intracellular ROS and antioxidants due to a sustained decrease in antioxidant capacity.

## 4 Conclusion

In this study, using IL‐1β‐stimulated chondrocytes and a DMM‐induced mouse OA model, we demonstrated that Ce@D&P NPs inhibit OA by exerting comprehensive antioxidant, iron‐chelating, and matrix repair functions. The research confirmed the multitarget regulatory effects of Ce@D&P NPs on ferroptosis. Therefore, Ce@D&P NPs holds significant potential for clinical application in the treatment of OA.

## 5 Experimental Section

### Materials

Deferasirox (CAS: 201530‐41‐8) was purchased from RHAWN (Germany). Pterostilbene (Cat: C12809247) and CeCl_3_ (Cat: C15135765) were obtained from Macklin Biochemical Technology Co., Ltd. (Shanghai, China). FerroOrange (Cat: F347) was purchased from DOJINDO (Kyushu, Japan). Antibodies against Col2A1 (A1560), GPX4 (A1933), ACSL4 (A20414) were obtained from ABclonal; Aggrecan (bs‐11655R), ADAMTS5 (bs‐3573R) from Bioss Biotechnology; MMP13 (ab39012) from Abcam. Ferritin (ET1610‐78) was obtained from HuaBio. Annexin V FITC apoptosis detection kit (556 547) was purchased from BD Pharmingen (USA). ROS detection kit (20101ES01) was purchased from Yeasen Biotechnology Co., Ltd. (Shanghai, China).

### Preparation of Ce@D&P NPs

First, D&P NPs were synthesized by linking deferasirox (DEF) and pterostilbene (PTE) through a thioketal linker. DEF and PTE were dissolved in anhydrous dichloromethane (DCM), followed by the slow addition of diisopropylethylamine (DIPEA) as a base under ice‐cold conditions. The reaction mixture was stirred continuously to facilitate the formation of the D&P nanoparticles. Subsequently, these D&P nanoparticles were subjected to micellization by adding the reaction mixture dropwise into distilled water under controlled ultrasonic conditions (60 W for 40 min at 0–4 °C). The reaction mixture was added at a rate of 1 mL min^−1^ while stirring at 500 rpm to ensure uniform distribution, promoting the self‐assembly of stable micelles. To enhance the antioxidant properties, cerium ions (Ce) were incorporated into the D&P nanoparticles using a coprecipitation method. Cerium chloride (CeCl_3_) was slowly added to the D&P nanoparticle suspension under constant stirring at room temperature, allowing the Ce ions to integrate into the nanoparticles. The reaction conditions, including the ratio of DEF to PTE and the amount of cerium ions, were optimized to achieve nanoparticles with an average size of ≈104.9 nm and a polydispersity index (PDI) of 0.077. After synthesis, the Ce@D&P nanoparticles were purified by high‐speed centrifugation to remove unreacted materials and solvents. The purified nanoparticles were then washed with anhydrous ethanol and distilled water, followed by vacuum drying to obtain the final Ce@D&P nanoparticle powder. The resulting nanoparticles were characterized using transmission electron microscopy (TEM) for morphology and particle size analysis to confirm their spherical structure and uniform distribution.

### Assessment of Cell Viability

Chondrocytes were seeded in 96‐well plates and cultured until reaching an appropriate density. Then, media containing different substances at specific concentrations, namely PTE, DEF, CeCl₃, and Ce@D&P NPs, were added, respectively, followed by further incubation for a certain period of time. Subsequently, CCK‐8 reagent was added to each well, and the plates were incubated in the dark. Finally, the absorbance of each well at a wavelength of 450 nm was measured using a microplate reader. Based on the absorbance values, the cell viability was calculated and the effects of these substances on the cells were analyzed.

### Cell Culture and Treatment

Under sterile conditions, the knee joints of mice were amputated with bone scissors and placed in sterile Petri dishes, washed with PBS solution. After removing the muscles and ligaments attached to the joints, the knee joints were cut into tissue fragments of ≈2–3 mm with ophthalmic scissors. Add 0.2% type II collagenase to the tissue and digest it at 37 °C for 4 h. After 4 h, the cell suspension was transferred to a 15 mL centrifuge tube and centrifuged at 1000 r min^−1^ for 5 min, and the supernatant was discarded. The cells were washed three times with PBS solution and filtered through a 200‐mesh cell strainer into a culture dish. Cells were cultured in DMEM high glucose medium containing 10% fetal bovine serum, 100 U mL^−1^ penicillin/streptomycin.

The cells were categorized into six groups based on the stimulation methods. The control group (con) was cultured with DMEM complete medium. The model group (vehicle) was subjected to modeling by adding 10 ng mL^−1^ IL‐1β and then PBS was dripped. For the deferasirox group (IL‐1β + 5 µm DEF), 5 µm DEF was added along with IL‐1β. In the pterostilbene group (IL‐1β + 5 µm PTE), 5 µm PTE was added together with IL‐1β. The CeCl3 group (IL‐1β + 100 µm CeCl_3_) had 100 µm CeCl3 added along with IL‐1β. And the Ce@D&P NPs group (IL‐1β + 2.5 µg mL^−1^ Ce@D&P NPs) involved adding 2.5 µg mL^−1^ Ce@D&P NPs along with IL‐1β. After 24 h of intervention, the cells were collected for subsequent experiments.

### Apoptosis Detection

Apoptosis was detected using the Annexin V‐FITC/PI kit following the manufacturer's instructions. Chondrocytes were incubated with Annexin V‐FITC and PI at 37 °C for 24 h, washed with PBS, centrifuged, and resuspended in 200 µL of binding buffer. After adding 10 µL of annexin V‐FITC solution and 5 µL of PI solution, the mixture was incubated for 15 min and then diluted with 300 µL of binding buffer. Apoptosis was analyzed by flow cytometry, defining the total apoptosis rate as the ratio of early apoptosis, late apoptosis, and necrotic cells to the total number of cells.

### ROS Detection

The intracellular ROS levels were detected using the cell‐permeable probe DCFH‐DA. Chondrocytes (1 × 10^4^ cells) were incubated in confocal dishes for 24 h, then incubated with 10 µm DCFH‐DA for 30 min and washed with PBS to remove unbound DCFH‐DA. ROS levels were measured by fluorescence microscopy. Additionally, some treated chondrocytes were collected, resuspended in DCFH‐DA, and incubated and washed thoroughly, then analyzed by flow cytometry at 488 nm wavelength to detect the number of positive cells.

### Assay for GSH/GSSG and MDA

Cortical GSH content was determined by a GSH and GSSG assay kit (S0053) following the provided guidelines of the manufacturer (Beyotime Biotechnology, Shanghai, China). The measurement was carried out on a colorimetric microplate reader at an optical density of 593 nm. The GSH content was presented in µmol g^−1^, and calculated for the test samples as: Total Glutathione – GSSG × 2. For cortical MDA content, a lipid peroxidation MDA assay kit (S0131S, Beyotime Biotechnology, Shanghai, China) was utilized according to the manufacturer's instructions. The ipsilateral cortex was first weighed and homogenized in chilled PBS on ice. Subsequently, the tissue lysates were centrifuged at 10 000–12 000 g at 4 °C for 10 min to obtain the supernatant. After adding 200 µL of MDA solution to each sample or standard (0.1 mL), the mixture was heated at 100 °C for 15 min and then centrifuged at 1000 g for 10 min to collect the supernatant. The supernatant (200 µL) was quantified colorimetrically, and MDA levels were expressed as µmol mg^−1^.

### Iron Ion Detection

FerroOrange solution was prepared by dissolving 24 µg of FerroOrange in 35 µL of dimethyl sulfoxide (DMSO), then diluting with Hanks' balanced salt solution (HBSS) solution to obtain a 1 µmol L^−1^ working solution. Treated cells were incubated with the working solution at 37 °C, 5% CO_2_ for 30 min and observed under a confocal microscope without washing.

### Western Blot

Cells treated in different groups were washed three times with PBS, then lysed with RIPA lysis buffer to extract total protein, and incubated on ice for 30 min. The total protein content was quantified using the BCA assay. Equal amounts of protein were separated by SDS‐PAGE, transferred to polyvinylidene fluoride (PVDF) membranes, and blocked with 5% skim milk at room temperature for 1 h. Membranes were incubated with primary antibodies overnight at 4 °C, followed by incubation with HRP‐conjugated secondary antibodies (1:5000) at room temperature for 1 h. Bands were visualized using an ECL kit and quantified with ImageJ software, normalized to GAPDH.

### RT‐qPCR

Total RNA was extracted from chondrocytes using TRIzol reagent (Invitrogen, USA). The RNA concentration and purity were determined by measuring absorbance at 260 and 280 nm. Complementary DNA (cDNA) was synthesized from 1 µg of RNA using the Revert Aid First‐Strand cDNA Synthesis Kit (Takara, Japan). Quantitative reverse‐transcription PCR (qRT‐PCR) was performed using the SYBR Premix Ex Taq Kit (Takara, Japan) and ABI 7500 sequencing detection system (Applied Biosystems, USA). The relative expression levels of GPX4, ACSL4, Col2, Aggrecan, MMP13, and ADAMST4 were calculated using the 2−ΔΔCT method, normalized to β‐actin. Experiments were conducted in triplicate for each condition. Primer sequences are provided in Table S1 (Supporting Information).

### Animals

Eight‐week‐old male C57BL/6 mice were obtained from the Experimental Animal Center of Zhejiang Chinese Medical University (Hangzhou, China). To avoid gender‐dependent differences, only males were used. The experimental animals were randomly sorted into six groups (*n* = 5 per group). The control group (con) was maintained under normal rearing conditions. The model group (vehicle) underwent DMM modeling and was administered an intra‐articular injection of 8 µL of normal saline. The deferasirox group was subjected to DMM modeling followed by an intra‐articular injection of 8 µL of 5 µm DEF. The pterostilbene group involved DMM modeling and an injection of 8 µL of 5 µm PTE. The CeCl_3_ group had DMM modeling and an injection of 8 µL of 100 µm CeCl_3_. And the Ce@D&P NPs group was modeled with DMM and then received an intra‐articular injection of 8 µL of 2.5 µg mL^−1^ Ce@D&P NPs. The injections were carried out once every 2 weeks. After 8 weeks, tissue samples were taken for further analysis. This study was approved by the Animal Ethics Committee of Zhejiang Chinese Medical University (Approval number: 20231016‐06).

### Construction of OA Mice Model

A destabilization of the medial meniscus (DMM) model was used to induce knee OA in 10‐week‐old male C57BL/6J mice, as previously reported. Briefly, under anesthesia, a 3 mm longitudinal incision was made on the medial side of the knee joint, and the medial meniscotibial ligament (MMTL) was exposed by blunt dissection of the knee extensor and patellar ligament. The MMTL was then transected to destabilize the medial meniscus (DMM). The medial joint capsule was sutured, and the skin was closed. Sham surgery was performed similarly without manipulating the MMTL.

### Micro‐CT

Eight weeks postsurgery, mice were euthanized, and the right knee joints were collected. Samples were fixed in 4% paraformaldehyde for 3 days and analyzed using microcomputed tomography (Micro‐CT, Skyscan 1176, Bruker µCT, Kontich, Belgium). The region of interest was the area between the proximal tibial growth plate and the tibial plateau. Data collected included 3D reconstructed images of the knee joint and the number of osteophytes.

### Histological Analysis

After Micro‐CT analysis, samples were decalcified in 14% EDTA for 14 days, dehydrated, and embedded in paraffin. Sections (4 µm thick) were stained with Alcian Blue Hematoxylin/Orange G and Hematoxylin & Eosin (H&E) to analyze cartilage structure changes. Cartilage structure degeneration was scored by three blinded observers following the recommendations of the International Osteoarthritis Research Society (OARSI). Scoring Range: 0: Normal cartilage with no signs of degradation. 1: Slight fibrillation of the articular cartilage surface, with minor superficial damage. 2: Small superficial erosions affecting up to 10% of the cartilage thickness. 3: Deeper erosions affecting up to 50% of the cartilage thickness, but less than 50% of the articular surface area. 4: Deep erosions affecting more than 50% of the cartilage thickness and more than 50% of the articular surface area, but with intact subchondral bone. 5: Severe cartilage destruction with subchondral bone exposure and possible osteophyte formation.

### Immunofluorescence

Chondrocytes were seeded in confocal dishes and incubated for 24 h under different conditions, then fixed with 4% paraformaldehyde. Cells were permeabilized with 0.5% TritonX‐100 and blocked with 5% bovine serum albumin for 20 min. Cells were incubated with primary antibodies (1:200) overnight at 4 °C, followed by incubation with Alexa Fluor488‐conjugated secondary antibodies at 37 °C for 1 h. Nuclei were stained with DAPI for 15 min, washed three times with PBS, and observed under a confocal microscope (LSM880, Zeiss, Germany).

### In Vivo Biosafety Evaluation

Mice were divided into experimental and control groups. Ce@D&P NPs were administered to the experimental group. After 8 weeks of treatment, the animals were sacrificed and the major organs (heart, liver, spleen, lung, kidney) were collected into 4% fixative solution. The tissues were paraffin‐embedded and cut into 3–5 µm sections. The sections were stained with hematoxylin for 3–5 min, washed, counterstained with eosin, dehydrated, cleared, and mounted. Finally, imaging was performed to observe the presence or absence of pathological changes.

### Transcript Profile Analysis

RNA sequencing libraries were prepared using the NEBNext UltraTM RNA Library Prep Kit for Illumina (NEB, USA) according to the manufacturer's instructions, with index codes added to each sample's attributes.

### Gene Expression Data Analysis

Differential expression analysis was performed using the DESeq2 R package (4.21), which uses a model based on the negative binomial distribution. The *p*‐values were adjusted using the Benjamini and Hochberg method to control the false discovery rate. Genes with a p‐value less than 0.05 detected by DESeq2 were considered as DEGs. We used the clusterProfiler R package to examine the statistical enrichment of DEGs in KEGG pathways, where a corrected p‐value less than 0.05 was regarded as significant enrichment of DEGs.

### Statistical Analysis

Statistical analyses were performed using GraphPad Prism 9 software for one‐way analysis of variance (ANOVA). The *t*‐test is used to determine whether there is a significant difference between the means of two independent groups. Data from at least three independent experiments were used, and results are presented as mean ± standard deviation (SD). Statistical significance was set at *p < 0.05, **p < 0.01, and ***p < 0.001.

## Conflict of Interest

The authors declare no conflict of interest.

## Supporting information



Supporting Information

## Data Availability

The data that support the findings of this study are available from the corresponding author upon reasonable request.
