# Supplementary material for: Nanodrugs Targeting Key Factors of Ferroptosis Regulation for Enhanced Treatment of Osteoarthritis
Source: Adv Sci (Weinh). 2025 Jan 22;12(11):2412817. doi: 10.1002/advs.202412817 (PMC11923906; doi:10.1002/advs.202412817)
Supplement: Supplementary file 1 — Supporting Information [file ADVS-12-2412817-s001.docx]

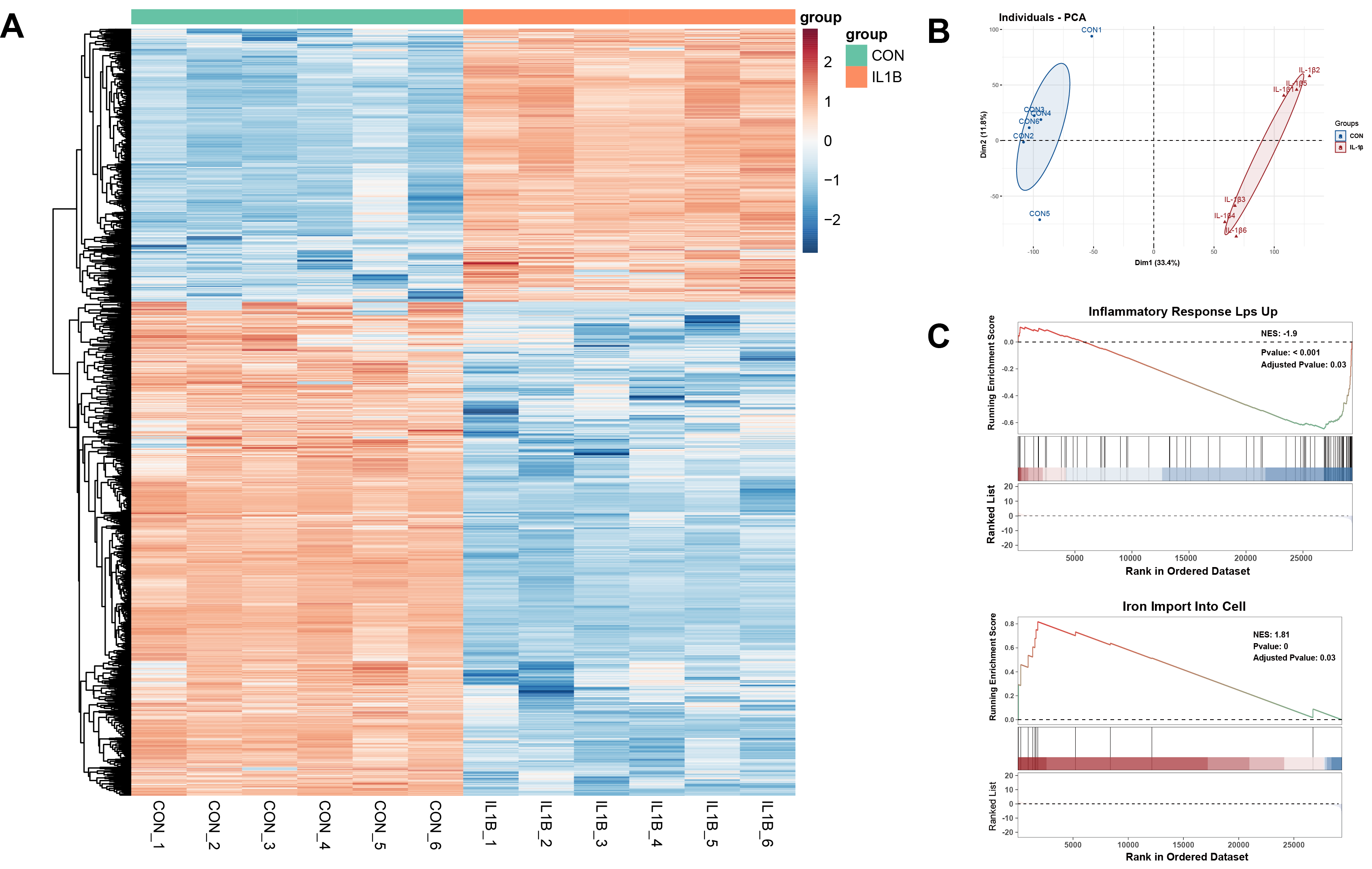


**Figure S1. (A)** Heatmap of DEGs in normal chondrocytes and IL-1β-stimulated chondrocytes (unmarked, n=6). **(B)** PCA of gene expression in normal and IL-1β-stimulated chondrocytes. **(C)** GSEA of DEGs between normal and IL-1β-stimulated chondrocytes.

**Figure S2.** Synthetic route of D&P NPs.


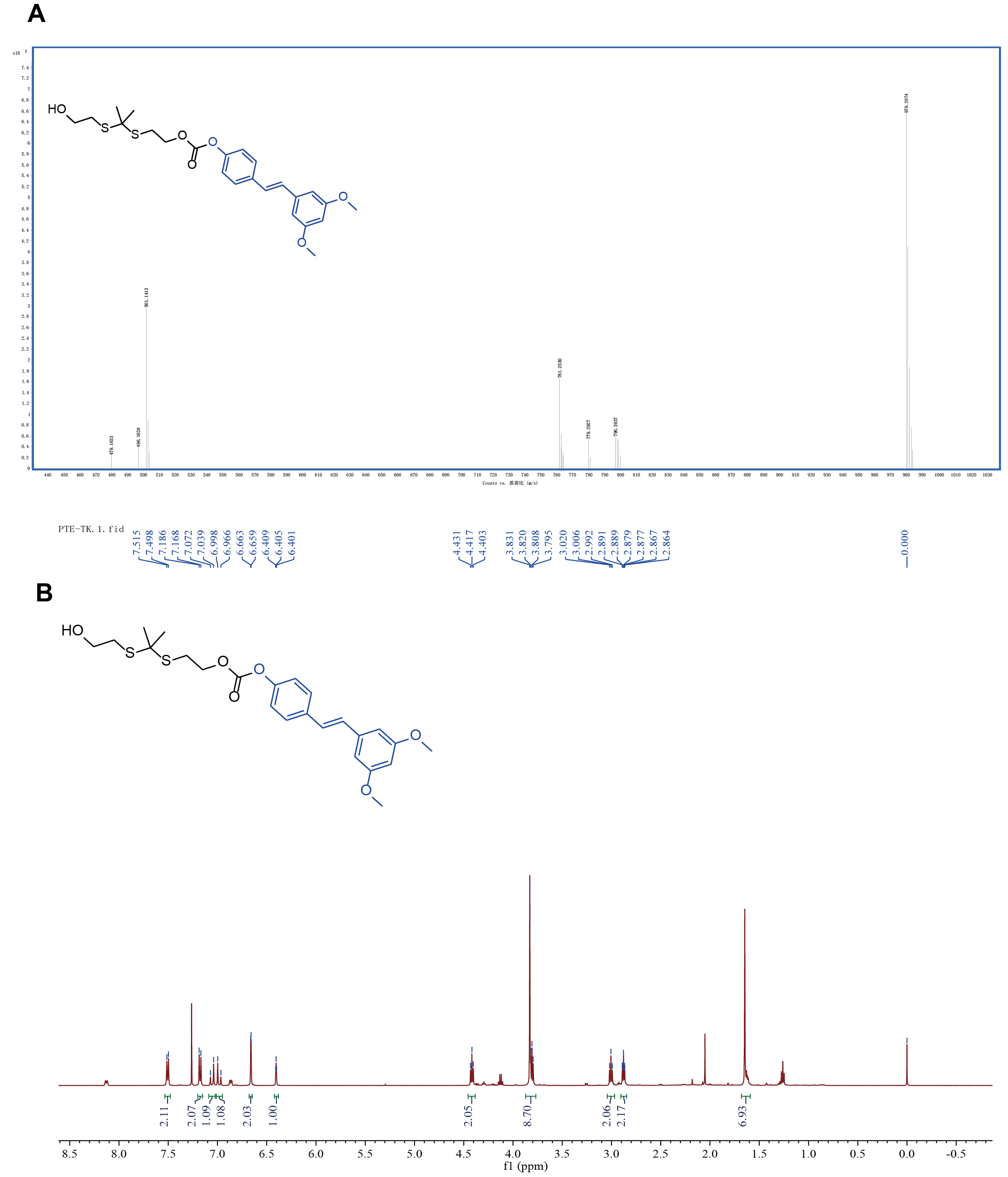


**Figure S3.** **(A)**^1^H NMR spectrum of DEF. **(B)** Mass Spectrum of DEF.


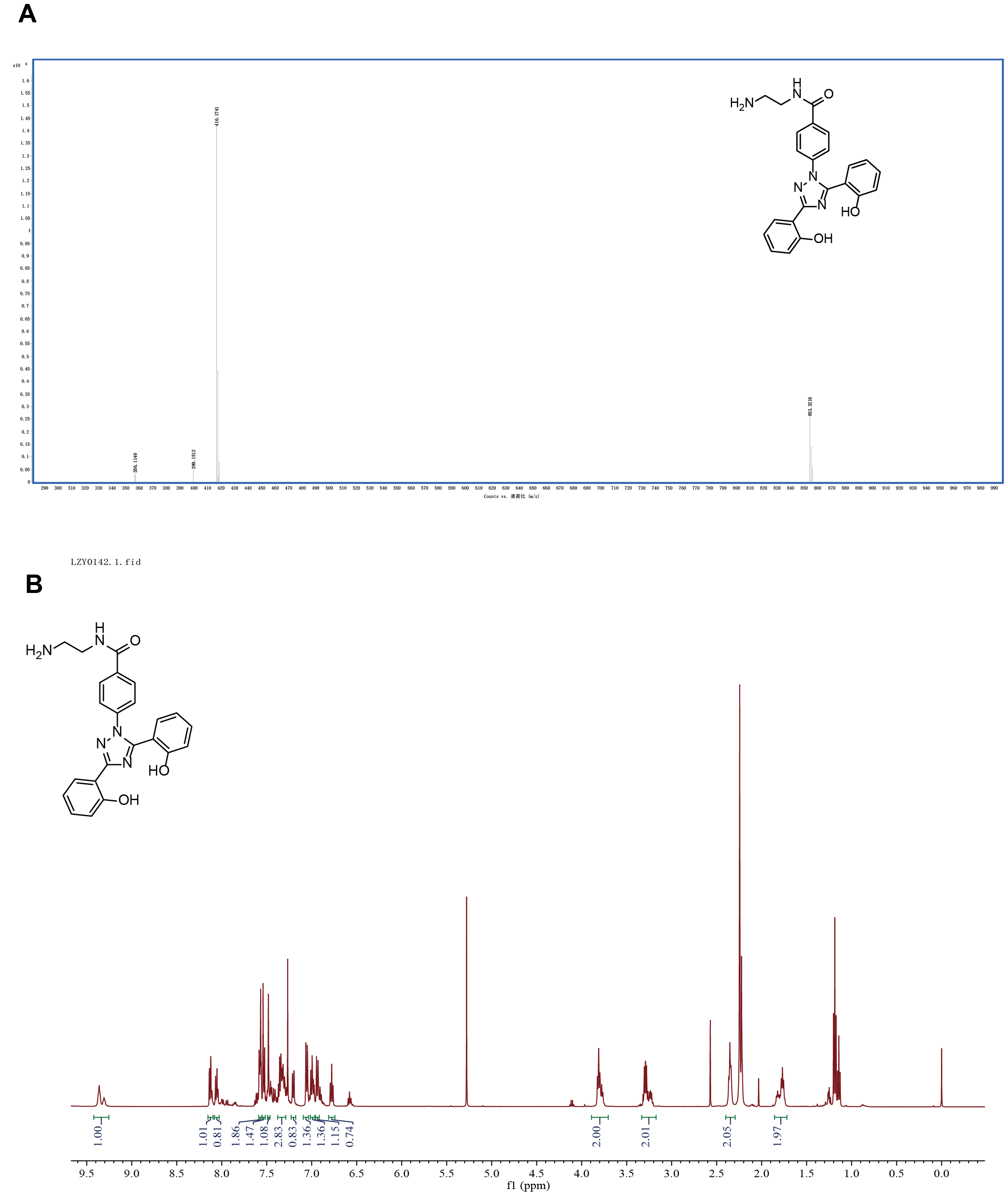


**Figure S4.** **(A)** ^1^H NMR spectrum of PTE. **(B)** Mass Spectrum of PTE.


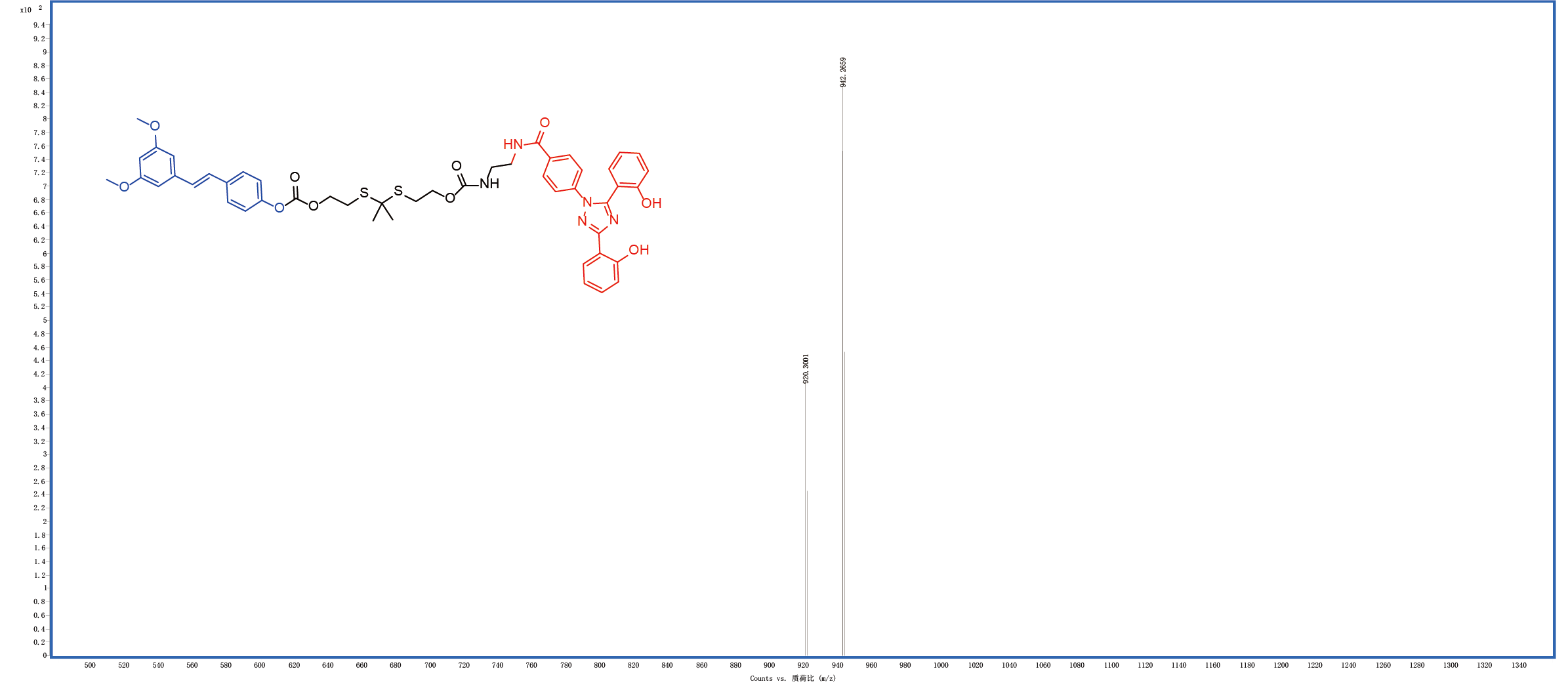


**Figure S5.** Mass Spectrum of D&P NPs.


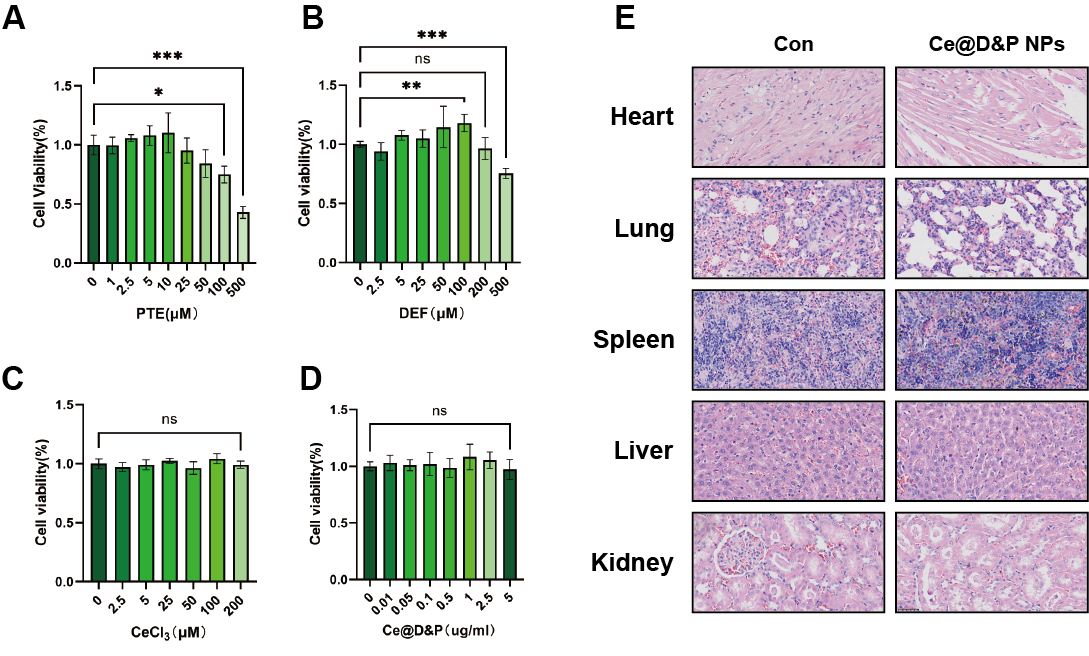


**Figure S6.** (A-D) The cell viability of chondrocytes after treatment with different concentrations of PTE, DEF, CeCl_3_ and Ce@D&P NPs. (E) The representative images of H&E - stained liver, kidney, spleen, heart, and lung sections from both mice treated with Ce@D&P NPs and control mice.


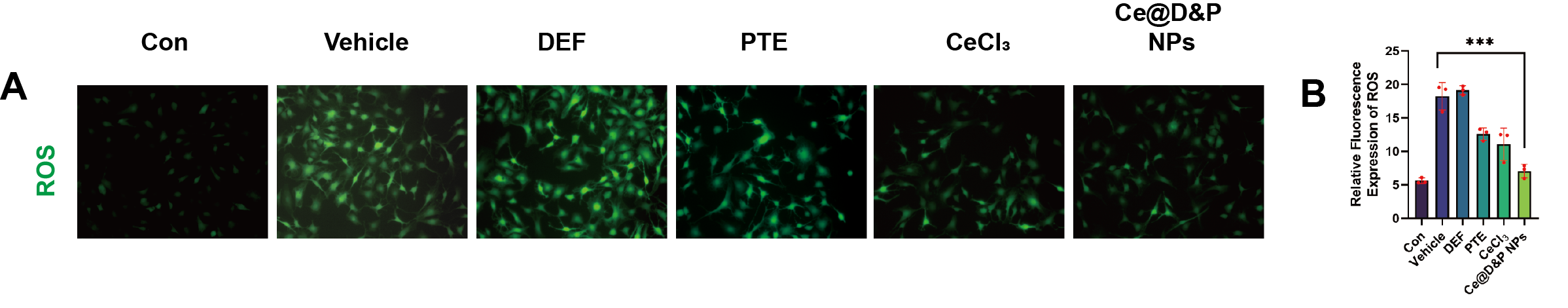


**Figure S7.** Detection and quantification of ROS-positive cells in chondrocytes by confocal fluorescence microscopy.


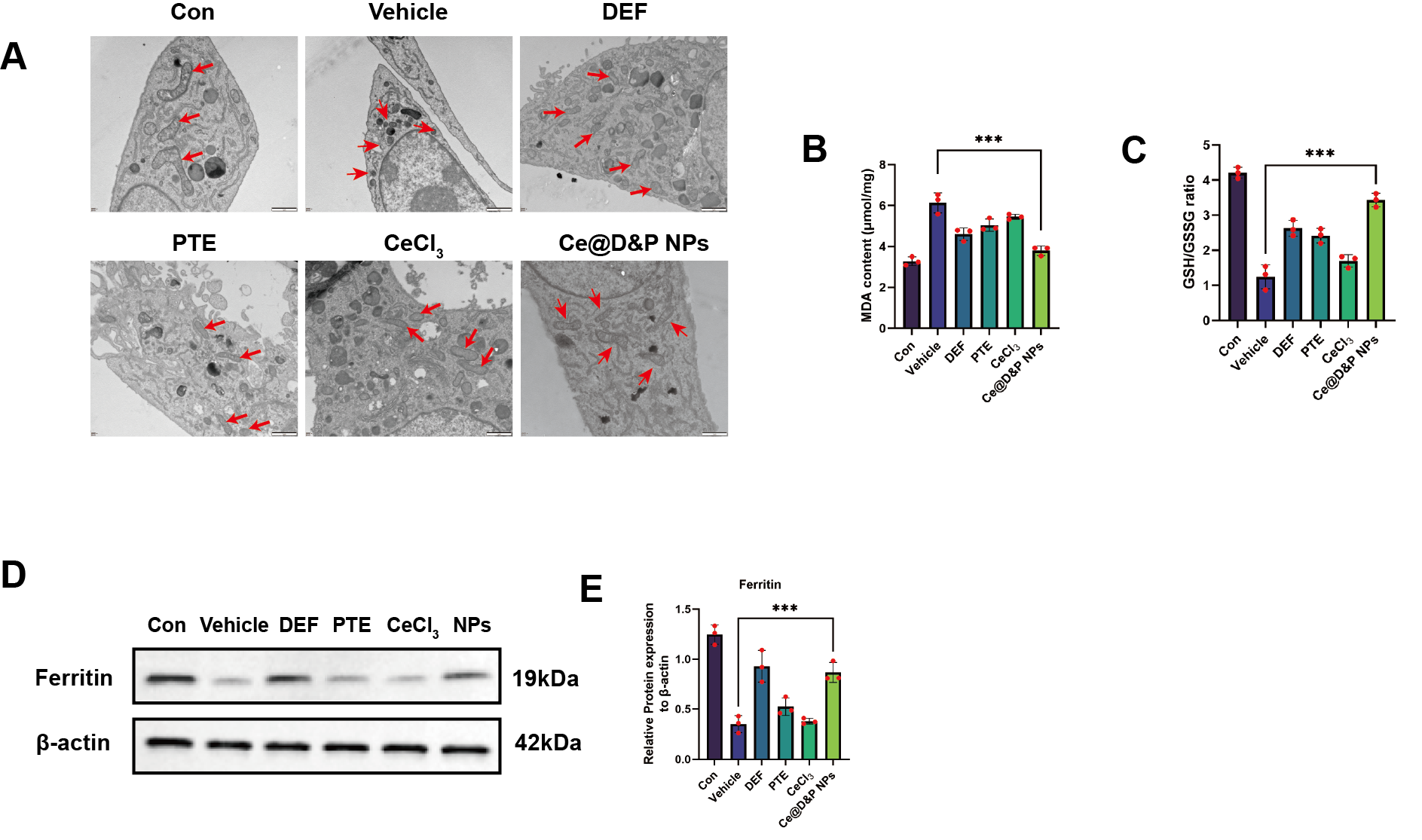


**Figure S8.** (A) Representative TEM images of chondrocyte mitochondria. Red arrows indicate mitochondria in chondrocytes. (B) The MDA content of the con group, vehicle group, and Ce@D&P NPs-treated group. (C) The GSH/GSSG ratio of the con group, vehicle group, and Ce@D&P NPs-treated group. (D) Western blot bands of Ferritin under different intervention conditions after IL-1β stimulation. (E) Relative protein expression levels of Ferritin.


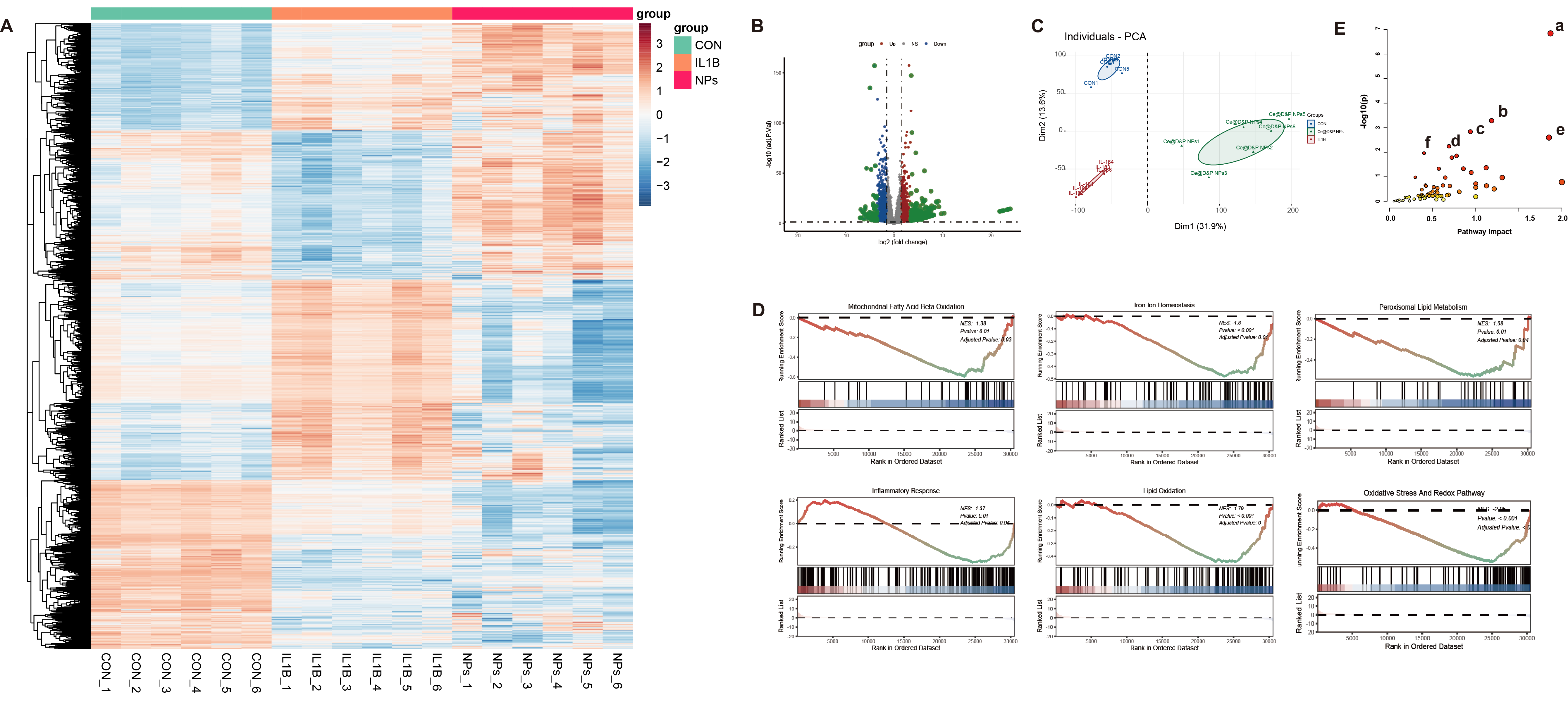


**Figure S9.** (A) Heatmap of DEGs between Ce@D&P NPs+IL-1β, IL-1β-stimulated vehicle group, and control group (unmarked, n=6). (B) Volcano plot showing the differential gene expression analysis between the IL-1β group and Ce@D&P NPs group. (C)PCA plot illustrating the distinct clustering of samples from the control, IL-1β, and Ce@D&P NPs groups based on gene expression profiles. (D) GSEA plots showing the enrichment of pathways related to mitochondrial fatty acid beta-oxidation, iron ion homeostasis, peroxisomal lipid metabolism, inflammatory response, lipid oxidation, and oxidative stress and redox pathways in chondrocytes treated with Ce@D&P NPs. (E) Pathway enrichment bubble plot illustrating the impact and significance of various metabolic pathways affected by Ce@D&P NPs treatment compared to the IL-1β group. Pathways are labeled (a. Purine metabolism; b. Pyrimidine metabolism; c. Biosynthesis of unsaturated fatty acids; d. Cysteine and methionine metabolism; e. Sphingolipid metabolism; f. TCA cycle) based on their impact.


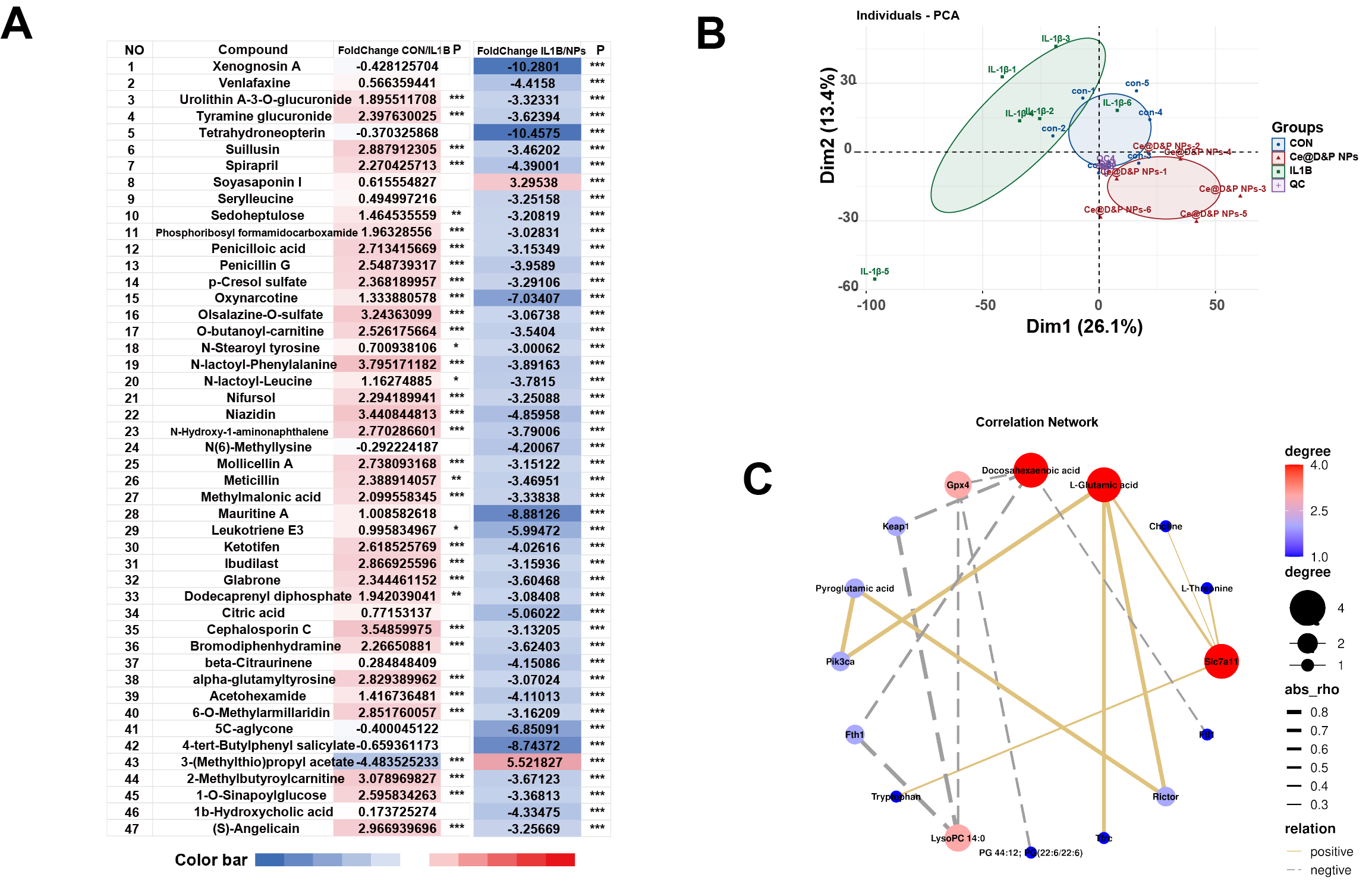


**Figure S10.** (A) Heatmap of significantly altered metabolites identified through metabolomic analysis, comparing the fold change in the IL-1β group versus the control group and the Ce@D&P NPs group. (B) PCA plot based on metabolomic data, showing distinct metabolic profiles of chondrocytes in the control, IL-1β, and Ce@D&P NPs treatment groups. (C) Correlation network diagram of differential genes and differential metabolites between the IL-1β group and the IL-1β+Ce@D&P NPs group.

**Table S1. RT–qPCR primers**

| **Primer** | **Primer sequence** **(5'to3')** | **Product length** |
| --- | --- | --- |
| Mouse GAPDH-F | GGTGAAGGTCGGTGTGAACG | 20 |
| Mouse GAPDH-R | CTCGCTCCTGGAAGATGGTG | 20 |
| Mouse GPX4-F | GATGGAGCCCATTCCTGAACC | 21 |
| Mouse GPX4-R | CCCTGTACTTATCCAGGCAGA | 21 |
| Mouse ACSL4-F | ATTGGTCAGGGATATGGGCT | 20 |
| Mouse ACSL4-R | AGAGGAGCTCCAACTCTTCCA | 21 |
| Mouse COL2-F | TGGTCCTCTGGGCATCTCAGGC | 22 |
| Mouse COL2-R | GGTGAACCTGCTGTTGCCCTCA | 22 |
| Mouse MMP13-F | TTTGAGAACACGGGGAAGA | 20 |
| Mouse MMP13-R | ACTTTGTTGCCAATTCCAGG | 20 |
| Mouse ADAMTS5-F | GGAGCGAGGCCATTTACAAC | 20 |
| Mouse ADAMTS5-R | CGTAGACAAGGTAGCCCACTTT | 22 |
| Mouse Aggrecan-F | GTGGAGCCGTGTTTCCAAG | 19 |
| Mouse Aggrecan-R | AGATGCTGTTGACTCGAACCT | 21 |
| Mouse TNF-α-F | ACCGTCAGCCGATTTGCTAT | 20 |
| Mouse TNF-α-R | CTCCAAAGTAGACCTGCCCG | 20 |
